# Does the Structure of Female Rhesus Macaque Coo Calls Reflect Relatedness and/or Familiarity?

**DOI:** 10.1371/journal.pone.0161133

**Published:** 2016-08-31

**Authors:** Dana Pfefferle, Kurt Hammerschmidt, Roger Mundry, Angelina V. Ruiz-Lambides, Julia Fischer, Anja Widdig

**Affiliations:** 1 Behavioural Ecology Research Group, Institute of Biology, University of Leipzig, Leipzig, Germany; 2 Junior Research Group of Primate Kin Selection, Department of Primatology, Max-Planck Institute for Evolutionary Anthropology, Leipzig, Germany; 3 Cognitive Neuroscience Laboratory, German Primate Center, Göttingen, Germany; 4 Leibniz-Science-Campus Primate Cognition, German Primate Center & Georg-August-University-Göttingen, Göttingen, Germany; 5 Cognitive Ethology Laboratory, German Primate Center, Göttingen, Germany; 6 Max-Planck Institute for Evolutionary Anthropology, Leipzig, Germany; 7 Caribbean Primate Research Center-Cayo Santiago, University of Puerto Rico, Puerto Rico, United States of America; University of Sussex, UNITED KINGDOM

## Abstract

In social animals, kin relations strongly shape the social structure of a group. In female-bonded species, maternal relatedness is likely to be mediated via familiarity, but evidence is accumulating that non-human primates are able to recognize kin that they are not familiar with and adjust their behavior accordingly. In playback experiments, female rhesus macaques showed increased interest in ‘coo’ calls produced by unfamiliar paternal half-sisters compared to ‘coo’ calls produced by unfamiliar unrelated females, suggesting that these calls should have some common structural characteristics that facilitate the discrimination of kin from non-kin. Here we analyzed ‘coo’ calls of 67 adult female rhesus macaques from four groups and seven matrilines living on the island of Cayo Santiago (Puerto Rico). We tested whether the call structure of closely maternal and/or paternal related females, as determined from extensive pedigree data, differed from the call structure of unrelated females, while controlling for familiarity (i.e., group-matrilineal membership and age difference) of subjects. In contrast to our expectation, kinship did not predict similarities in ‘coo’ call structure, whereas ‘coo’ structure was more similar when produced by females of similar age as well as by females with higher familiarity, suggesting that experience is more decisive than genetic background. The high number of individuals in the analysis and the high accuracy of the assignment of calls to individuals render a lack of power as an unlikely explanation. Thus, based on the results of this study, kin recognition in rhesus monkeys does neither appear to be based on the assessment of self-similarity, nor on the comparison among related subjects (i.e., acoustic phenotype matching), but appears to be mediated by different or multiple cues. Furthermore, the results support the notion that frequent social interactions result in increasing acoustic similarity within largely innate call types (‘vocal accommodation’).

## Introduction

Gregarious primates form differential relationships across group members, with some of those relationships being very close and enduring, persisting for months or even years [1]. Kinship is one of the factors influencing the formation of those relationships [2], facilitating the acquisition of inclusive fitness benefits [3] via nepotism (i.e., the preferential treatment of close relatives in pro-social interactions [4]) and/or optimal outbreeding (i.e., the balancing of fitness benefits due to mating with close kin against the costs of inbreeding depression [5]).

A prerequisite of kin selection is that kin must be sufficiently distinctive from non-kin, as well as recognized and preferentially treated. Consequently, mechanisms and cues facilitating these processes should have evolved. Among the most likely mechanisms used to identify kin are familiarity through prior association and phenotype matching in which a target phenotype is compared with a template derived from either oneself or a known relative [6].

Like in other mammalian species, the disproportional investment of non-human primate mothers into their offspring leads to a system in which maternal kinship and familiarity are particularly closely related. It is hence not surprising that familiarity can be invoked to explain most reports on maternal kin discrimination found in today's literature on non-human primates (e.g., [7–10]).

Disentangling familiarity and phenotype matching is challenging as it requires demonstrating kin recognition (i.e., the ability to identify, distinguish, and classify kin vs. non-kin [11]) or kin biased behaviors (i.e., the differential treatment of kin and non-kin [11]) while controlling either for familiarity or phenotype matching. Under natural conditions, it is difficult to eliminate phenotype matching as an underlying mechanism, as this would require precluding any learned or genetically based kin template. Two circumstances favor the existence of unfamiliar (i.e., paternal) kin in order to control for familiarity under natural conditions. First, if male annual reproduction is skewed toward one or a few males within one social group, these offspring will be paternal half-siblings born to different mothers. Hence, paternal siblings will be less familiar than maternal siblings as they live in different social environments compared to maternal siblings, which are familiar through their common mother. Second, if males disperse at least once in their life while reproducing in more than one group, these paternally related individuals will be even less familiar to each other than those born in the same group, particular when considering the philopatric sex (in this case females), as they can be expected to never reside in the same group. Taking advantage of such a situation, studies on two primate populations have been able to provide evidence for phenotype matching among unfamiliar kin (rhesus macaques, *Macaca mulatta*: [12,13]; mandrills, *Mandrillus sphinx*: [14]).

Among the cues facilitating kin recognition are olfactory, visual and auditory signals (reviewed in [15,16]). For example, there is evidence that acoustic features vary with regard to relatedness (based on kin [17–19], based on maternal and paternal kin [14]). The logic behind greater acoustic similarity with increasing relatedness is that kin may share specific features of their vocal pattern generators and/or morphological features in their sound apparatus affecting acoustic characteristics of the produced vocal signal [20]. The challenge is to distinguish acoustic similarity due to genetic relatedness from acoustic similarity which may arise out of experience [21,22]. Although the acoustic structure of non-human primate vocalizations appears largely innate, exposure to specific sound characteristics of others may lead to an increasing acoustic similarity between subjects, a phenomenon termed ‘vocal accommodation’ [23]. Vocal accommodation requires only little articulatory control because it affects mainly the phonation qualities, like pitch, loudness and duration of utterances [24,25].

Studies on acoustic similarities that consider familiarity and genetic relatedness between individuals are rare. Calls of Campbell’s monkeys (*Cercopithecus campbelli campbelli*) have been found to be more similar among more strongly bonded animals (measured by grooming duration), independent of genetic relatedness [26]. In contrast, a recently published study on mandrills reported an effect of both, kinship and familiarity, on contact call characteristics [14]. Results of playback experiments, testing the effect of kinship and familiarity on the response of conspecifics, support the finding of genetic relatedness being reflected in the acoustic structure of contact calls (rhesus macaque females: [12]; mandrill males and females: [14]).

Taking advantage of the demographically well monitored (nearly daily census records since 1956) and, in terms of pedigree information (genetic database implemented in 1992), most comprehensive rhesus macaque population on Cayo Santiago (Puerto Rico, USA), we set out to investigate whether information about relatedness is reflected in the acoustic structure of their contact calls (‘coo’). Our dataset consisted of acoustic information for 67 adult females that were either unrelated, patrilineal kin, matrilineal kin, or dyads related via both the maternal and paternal kin-lines. In addition to information about relatedness, we included information on the degree of familiarity between individuals. As measures of familiarity we used group membership, matrilineal membership, and age difference. In order to distinguish same vs. different matrilines it is helpful to go back several generations to avoid misclassifications. In our study population, information on matrilineal membership dates back to the very first females that founded the colony about 70 years ago. Therefore, some females, although in the same matriline, are related at a very low degree (r < 0.0625) (see methods for our definition of kin and non-kin dyads). To examine the effect of familiarity, we used the interaction between group membership, matrilineal membership, and age difference, as we assumed that individuals of the same group and matriline, as well as peers of the same group, interact preferentially with each other [10,27,28].

In accordance with previous findings, we predicted genetic relatedness and/or our proxies of familiarity (i.e., age difference, group- and matrilineal membership) to affect acoustic similarity between individuals in a way that more familiar and more closely related individuals show greater similarities (or smaller acoustic distance) in the acoustic structure of their contact calls.

## Methods

### Study site and subjects

We conducted the study on the rhesus macaque population living on Cayo Santiago, a 15.2 ha island offshore of Puerto Rico. Rhesus macaques live in female bonded, multi-male-multi-female groups, with males dispersing on average at 5 years of age [29]. Information on date of birth, natal group and duration of group membership of all animals were available from the demographic database of the Caribbean Primate Research Center (CPRC). This database is based on records of nearly daily censuses conducted continuously since 1956. All individuals were habituated to human observers and could be recognized on an individual basis using tattoos, ear notches or individual characteristics. During data collection (April to December 2009 and March to August 2010) approximately 1000 individuals belonging to 6 different social groups inhabited the island, with group sizes ranging from approximately 100 to 300 individuals. Data reported here stem from 67 adult females (age range: 4 to 24 years, mean: 9.6 years) belonging to 4 groups and 7 matrilines (mean = 2.75 matrilines/group, range: 1–4, mean number of females within matrilines = 62.27, based on census October 2009).

### Vocal recordings

**Fig 1:**
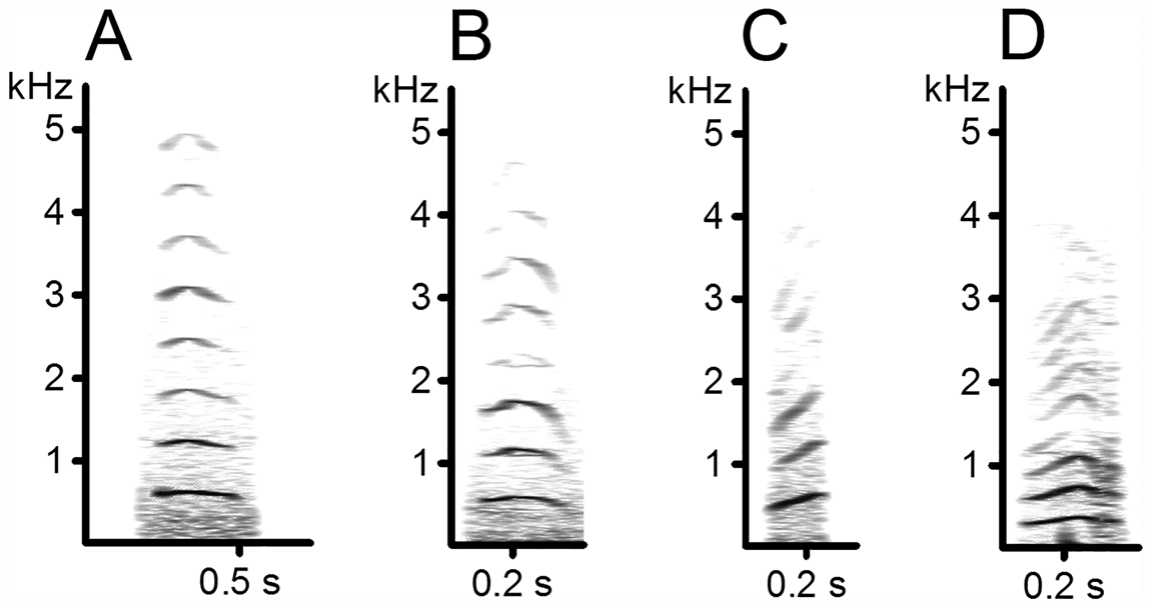
Spectrograms of rhesus female coo calls. Coo call of subject A and B have a relatively high similarity (i.e., a low F-value of 0.97), whereas the coo calls of subject C and D have a low similarity (high F-values of 6.71). F-values for the other combinations: A vs. C = 4.5, A vs. D = 3.7, B vs. C = 4.63, B vs. D = 3.81.

We recorded ‘coo’ calls uttered in the same behavioral context (i.e., group progression [7], see Fig 1A–1D). Vocalizations were recorded *ad libitum* throughout the day using a Marantz PMD 661 recorder (D & M Professional, Longford, U.K.) and a Sennheiser directional microphone (Sennheiser, Wedemark, Germany; K6 power module and ME66 recording head with Rycote Modular Windshield System and a Rycote Windjammer, Rycote Ltd., Stroud, U.K.). Rhesus macaques utter ‘coo’ calls as single calls as well as in bouts. For analysis, we only selected one call per bout, or calls that were uttered singly. Because the macaques on Cayo Santiago are well habituated, including to the use of acoustic recording equipment, we could make recordings at very close range (mean ± SD: 1.79 ± 1.05 m). Recordings were transferred to a computer and saved at 16-bit accuracy and a sampling frequency of 44.1 kHz. Only high quality recordings, i.e., not disturbed by background noise, were selected and entered into our structural analysis. In total 620 ‘coo’ calls of 67 females (14 to 4 per individual, mean = 9.2) entered the acoustic analysis.

We acknowledge that it would have been interesting to also investigate structural similarities between male contact calls. This was, however, not possible, because males emitted fewer ‘coo’ calls than females (Pfefferle et al. unpublished data) providing us with an insufficient number of appropriate vocalizations.

### Parentage assignment

For parental assignment we used the long-term genetic database for this population, currently encompassing 3735 individuals (details in [12,30]). In brief, blood samples were collected from most animals of the population with extensive sampling efforts since 1992. Genetic sampling of individuals is routinely conducted by the CPRC during annual trapping. Animals are trapped and anesthetized using an intramuscular injection of Hydrochloride Ketamine (10 mg/kg body weight), and blood samples are drawn via femoral venipuncture by CPRC veterinarians. Two samples with a maximum of 2 ml of blood each were obtained (for more details [30]).

We analyzed data from 67 females, whose mothers (N = 49 unique mothers, as some subjects shared the same mother) listed in the demographic database were genetically confirmed. Maternity information was used in subsequent paternity analyses, for which all males older than 1250 days (based on earliest age at reproduction [31]) and present on the island at least 200 days prior to the birth of a given individual (based on mean days ± SD gestation length: 166.5 ± 7.4; [32]) were considered as potential sires. On average mother-sire-offspring trios were typed on 29 common loci. Paternity was assigned for all 67 females using a combination of exclusion and likelihood methods. In all cases the assigned sire had no mismatch with the respective mother-offspring pair while all other potential sires were excluded by at least two loci, resulting in a total of 38 unique sires. Relatedness between individuals was additionally confirmed at 95% confidence level by the maximum likelihood method (CERVUS 3.0, [33]) for all, but five cases for which relatedness was confirmed at 80% confidence level.

To calculate the degree of relatedness among dyads, we also aimed to assign the maternal and paternal grandparents. For 41 of the 49 unique dams we could determine a mother (N = 28 unique identities), i.e., maternal grandmothers of the offspring, confirming the demographic data (i.e., nearly daily observations by the CPRC from birth until weaning) in all cases. In the remaining cases (N = 8) no genetic sample from the maternal grandmother was available. Based on the 38 unique sires, we genetically identified 23 unique paternal grandmothers. In 15 cases no genetic sample from the paternal grandmother was available. However, given that in the entire study population only 80 (2.47%) of all behaviorally-determined mothers (N = 3247) were not subsequently confirmed genetically, we feel confident in using the behaviorally-assigned (grand)mothers in these cases. Based on the 49 unique dams, we could determine 31 unique maternal grandfathers, excluding all other potential grandfathers at two or more loci. Relatedness between dams and maternal grandfathers was confirmed at 95% confidence level by the maximum likelihood method (CERVUS 3.0, [33]) for all, but one case for which relatedness was confirmed at 80% confidence level. For the 38 unique sires, we identified 23 unique paternal grandfathers, excluding all other candidates by at least two mismatches. Relatedness between sire and paternal grandfather was confirmed at 95% confidence level using the maximum likelihood method (CERVUS 3.0, [33]) for all, but one case for which relatedness was confirmed at 80% confidence level.

### Determination of the degree of relatedness

Pedigree information up to the grandparental generation was subsequently used to calculate the degree of relatedness (r) between dyads (N = 2178 dyads based on 67 individuals). For those individuals having one or more ancestors in common, we added relatedness from the maternal and paternal linages. In order to be defined as a kin dyad [r = 0.0625–0.5] (i) at least 10 of the 12 possible ancestors (i.e., 2 pairs of parents plus 4 pairs of grandparents) had to be identified, and (ii) at least one of the ancestors had be shared. This definition was met for 307 dyads (hereafter ‘kin dyads’, N = 134 with 12, N = 101 with 11, and N = 72 with 10 known ancestors, for information about the distribution of r-values see Table 1). It is important to note that r-values reflect the minimum value of relatedness, i.e., a dyad may have been more (but not less) closely related than indicated by our r-value (see [34]). In contrast, to be defined as a non-kin dyad, we stipulated that up to the grandparent generation i) all 12 ancestors of a given dyad had to be identified and ii) none of them had to be shared by the individuals constituting the dyad (i.e., non-kin dyads shared no ancestor up to the grandparent generation). For those dyads (N = 726) we assumed that the grandparents in the pedigree were themselves unrelated to one another. If this assumption would be incorrect and two grandparents were related at r = 0.5 (i.e., the highest probable level), we would have had underestimated the level of kinship in this dyad by r = 0.03125, creating only minor noise in the kinship values [34]. Only dyads that met either our kin or non-kin category entered the data set for the final analyses, which consisted of 1033 dyads of 67 females (i.e., N = 1145 dyads had to be excluded per definition above).

**Table 1:** Number of dyads with respective relatedness values in the three defined categories.

r-value	same group, same matriline	same group, different matriline	different group, different matriline
0	296	181	249
0.0625	69	24	7
0.1250	80	23	7
0.1875	4	0	0
0.2500	45	15	5
0.3125	4	1	0
0.3750	2	0	0
0.5000	21	0	0

### Structural analysis of ‘coo’ calls

To obtain an appropriate range for the estimation of acoustic features of ‘coo’ calls with an improved frequency resolution, we reduced the sampling frequency from 44.1 to 11.05 kHz and calculated a 1024 pt Fast Fourier Transformation (FFT) (frequency range = 5.5 kHz, frequency resolution = 11 Hz, time resolution = 2.9 ms). We submitted the resulting frequency time spectra to the custom software program LMA 2010 (developed by K. Hammerschmidt [35]) that extracts different sets of features from acoustic signals.

To estimate the individual specificity of ‘coo’ calls we calculated the following acoustic features: First, we used an autocorrelation function for each time segment (time frame of FFT) in a given ‘coo’ call to estimate the fundamental frequency (F0) and features related to F0. Depending on the number of peaks and the periodicity of the autocorrelation function each time segment was classified as noisy (no peaks could be detected), complex (some peaks could be detected, but they were not periodic), or tonal (periodic peaks). If a time segment was classified as tonal we determined the F0. We visually controlled the results via a harmonic cursor method implemented in LMA 2010 [36]. In addition to F0 and tonality we calculated features describing the harmonic-to-noise ratio (HNR) and peak frequency (PF) from the tonal time segments. Second, to describe the general energy distribution of single ‘coo’ calls we calculated the statistical distribution of frequency amplitudes across the spectrum. In a first step we calculated the overall energy of each time segment of a call. Subsequently, we determined the frequency at which the distribution of the amplitude in the frequency spectrum reached the first, second, and third quartile of the total distribution. In this way we were able to describe energy changes within a call independent of structural assumptions. Furthermore, we characterized the main energy of the entire ‘coo’ calls by calculating the peak frequency (i.e., frequency of highest amplitude), frequency range and global energy peaks within the frequency spectra [36].

To estimate the relationship between the individual acoustic structure of their ‘coo’ calls and their genetic relatedness we used the pairwise distances of a stepwise discriminant function analysis given as F-values (DFA, SPSS19). This method has been applied in different studies examining the relationship between acoustic structure and genetic or geographic distance of populations [37–39]. In a first step, the stepwise procedure identifies the combination of acoustic features that allows an optimal discrimination between all subjects by excluding all acoustic parameters that do not improve the discrimination (correlating parameters with no additional explanatory value). This also leads to a reduction of collinearity of acoustic features. In a second step, the discriminant function estimates the F-values of pairwise distances between individuals. To estimate the similarity between individuals, we entered all 112 acoustic features into a stepwise DFA, using subject identity as grouping variable. The selection criterion for acoustic features was to enter them in the discriminant function when p ≤ 0.05, and remove them when p ≥ 0.1. We used the F-values of the last step to describe the similarity between female ‘coo’ calls.

### Statistical analysis

To analyze whether the acoustic distance (expressed as F-values of the DFA, see above) between individuals was influenced by their degree of relatedness, we fitted a linear mixed model (LMM) [40]. We controlled for different levels of familiarity by including group membership (born into same vs. different group), matrilineal membership (born into same vs. different matriline) and age difference (a continuous variable indicating the birth difference in years) as predictors into our model.

Due to the dependency of group- and matrilineal membership, we merged these two factors into one comprising three levels: different group and different matriline, same group and same matriline, and same group but different matriline. Hereafter, this factor is called ‘group-matrilineal membership’. Based on previous evidence, we predicted that relatedness and/or familiarity influenced acoustic distance; hence, we assumed an interaction between the degree of relatedness and familiarity (i.e., group-matrilineal membership and age difference). Therefore, we included the three-way interaction between these three predictors (and also all two-way interactions this encompasses) into our full model. To keep type I error rates at the nominal level of 0.05, we added all possible random slopes [41,42], including those accounting for the interactions, to the model. To include random slopes for the factor group-matrilineal membership we manually dummy coded it and entered the two derived dummy variables and also their products with the respective other terms (to include random slopes for the interactions) into the random slopes components of the model. However, we did not include the correlations between random slopes and random intercepts in order to reduce computation time and since it is known that neglecting them does not compromise type I error rates [42]. As random effects, we entered the identities of the two subjects of a dyad. Prior to fitting the model we checked all predictors, as well as the response (i.e., acoustic distance) for their distribution and, as a consequence, square root transformed age difference and acoustic distance to achieve a more symmetrical distribution, as well as normally distributed and homogeneous residuals (verified by visual inspection of a qq-plot of the residuals and residuals plotted against fitted values). Afterwards we z-transformed all continuous predictors (i.e., degree of relatedness and age difference) to a mean of zero and a standard deviation of one to achieve comparable estimates and a more easily interpretable model with regard to the interactions [43,44]. The model was fitted in R v.3.2.0 using the function ‘lmer’ provided by the package 'lme4' [45]). We checked for model stability by excluding subjects one at a time from the data and comparing the estimates derived for the obtained data sets with those obtained from the model based on all data. This indicated that no heavily influential case existed. Additionally, we randomized the assignment of subjects to the two random effects and compared the range of the estimates derived from the random assignments. This revealed some moderate uncertainty in the case of the full model, namely for the three-way interaction, but the final model appeared to be robust (see below). Generalized Variance Inflation Factors (VIF, [46,47]) were derived using the function ‘vif’ of the R-package car [48] applied to a standard linear model excluding the interactions and random effects. Results indicated no collinearity issue (largest VIF = 1.1; [46,49–51]).

As an overall test of the effect of the predictors (degree of relatedness, age difference, group-matrilineal membership, and their interactions) on the acoustic distance [52], we used a likelihood ratio test (LRT) comparing the full model (including all interactions) with a null model comprising only the intercept and the random effects using the R function ‘anova’ with argument test set to ‘Chisq’. Since we were particularly interested in the outcome of the three-way interaction (see our prediction above), we first inspected the results of the full model. Because the interpretation of the main effects is only possible after removal of all non-significant interaction terms, we checked whether the model including the three-way interaction term differed from the model without this three-way interaction using LRT. We then removed any non-significant interactions, to infer about the results for the main effects. Our dataset included 1033 dyadic similarity measures with dyads being composed of a total of 67 individuals.

### Ethics statement

This study was approved and carried out in strict accordance to the rules and requirements of the Caribbean Primate Research Center and the Institutional Animal Care and Use Committee, Medical Sciences Department, University of Puerto Rico (protocol No. 4060105).

## Results

The stepwise parameter selection of the DFA resulted in the inclusion of 49 (out of 112) acoustic features for an optimal individual differentiation. With these features, calls could be assigned to the correct individual (N = 67) in 68.5% of cases (chance level = 1.49%, approx. 50 times above chance), indicating a high degree of individual differentiation. The five most important acoustic features to describe similarity of coo calls, and, hence, to discriminate between rhesus macaque females, were: mean F0, call duration, maximum F0, amplitude ratio between F0 and 3^rd^ harmonic, and the 3^rd^ quartile of the distribution of frequency amplitudes. Importance and description of all 49 acoustic features is given in S1 Table. Fig 1A–1D shows spectrographic examples of female coo calls with high and low structural similarity.

**Fig 2:**
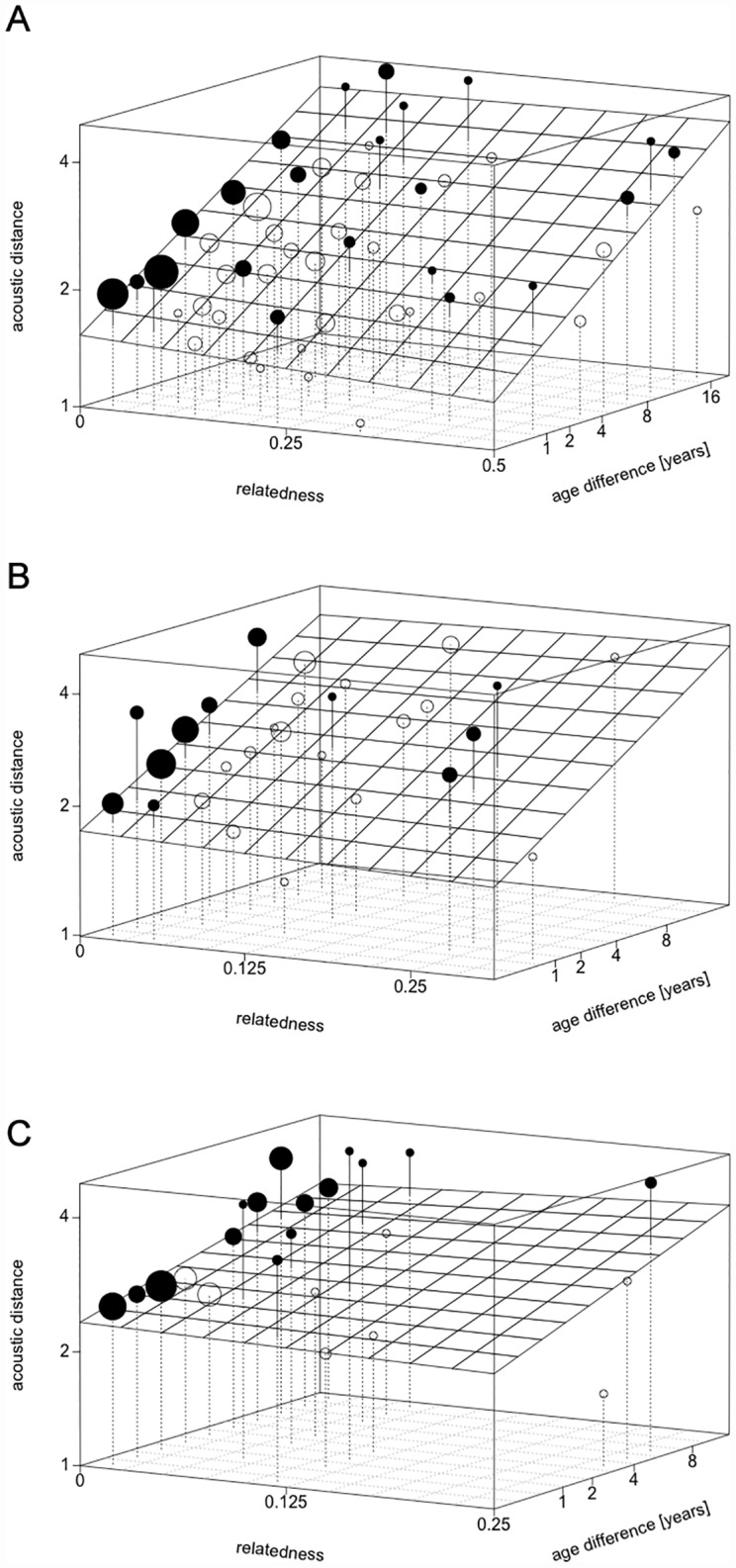
Acoustic distance vs. relatedness and age difference, plotted for the three levels of group-matrilineal membership. Acoustic distance plotted against relatedness and age difference for the three different levels of group-matrilineal membership: (A) same group & same matriline, (B) same group & different matriline, (C) different group & different matriline. The grid indicates the predictions of the model, while circles indicate actual data points. The area of a circle represents the number of data points plotted, and the color indicates whether the data points fall above (black) or below (white) model prediction.

We found strong evidence that the full model differed from the null model (LRT comparing the full with the null model: Χ^2^ = 67.54, P < 0.001, df = 11), suggesting that the set of predictor variables had a clear influence on the acoustic distance (see Table 2). Fig 2A–2C shows the acoustic distance in relation to relatedness and age difference for the three levels of group-matrilineal membership. The corresponding three-way interaction between relatedness, group-matrilineal membership and age difference revealed no marked effect on the acoustic distance of rhesus ‘coo’ calls (LRT: Χ^2^ = 0.454, df = 2, P = 0.797). Furthermore, none of the three two-way interactions had a substantial effect on acoustic distance (relatedness:group-matrilineal membership LRT: Χ^2^ = 0.252, df = 2, P = 0.882; relatedness:age difference LRT: Χ^2^ = 1.382, df = 1, P = 0.240; group-matrilineal membership:age difference LRT: Χ^2^ = 0.543, df = 2, P = 0.76).

**Table 2:** Results of the full model testing the effect of relatedness, matrilineal-group membership and age difference on acoustic distance between dyads.

	Estimate	SE	t-value
(Intercept)	1.403	0.054	26.019
relatedness	-0.020	0.037	-0.556
same group-same matriline	-0.116	0.046	-2.511
same group-different matriline	-0.054	0.047	-1.130
age difference	0.190	0.037	5.079
two way interactions:			
relatedness: same group-same matriline	0.003	0.038	0.081
relatedness: same group-different matriline	0.016	0.043	0.359
relatedness: age difference	0.039	0.045	0.880
same group-same matriline: age difference	0.011	0.038	0.295
same group-different matriline: age difference	0.012	0.040	0.296
three-way interaction:			
relatedness: same group-same matriline: age difference	-0.030	0.045	-0.661
relatedness: same group-different matriline: age difference	-0.024	0.054	-0.453

**Fig 3:**
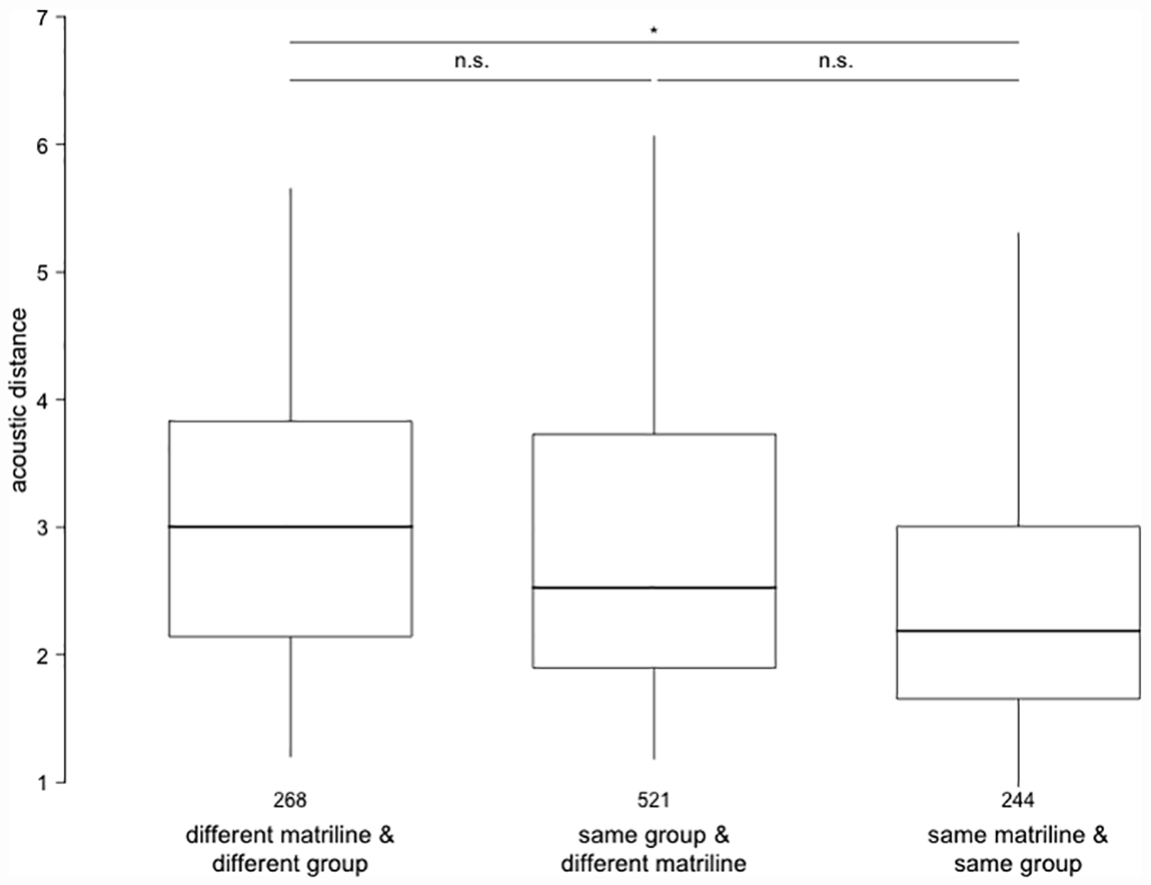
Acoustic distance vs. group-matrilineal membership. Acoustic distance plotted for the three levels of the factor group-matrilineal membership, i.e., dyads of individuals of different matriline and different groups, same matriline and same group, same group, but different matriline. Results of the post-hoc comparison between the three levels indicated by ‘n.s.’(p > 0.05) or * (p < 0.001). Numbers above the x-axes labels indicate the number of dyads represented in the respective level. The horizontal lines represent the median, boxes the quartiles and vertical lines depict the percentiles at 2.5% and 97.5%.

The inspection of the retained main effects yielded no effect of relatedness on acoustic distance (Table 3). Group-matrilineal membership, however, influenced acoustic distance such that dyads that lived in the ‘same group and same matriline’ revealed a significantly smaller acoustic distance than dyads living in ‘different groups and different matrilines’ (Table 3, Fig 3). The acoustic distance of dyads living in the ‘same group and different matriline’ fell in between the two other categories, such that the characteristics of dyads living in the ‘same group and the same matriline’ were not significantly different from dyads living in the ‘same group and different matriline’ (Table 3, Fig 3). Likewise, dyads from ‘different groups and matrilines’ did not differ significantly in their acoustic distance from dyads living in the ‘same group and different matrilines’ (Table 3, Fig 3).

**Table 3:** Results of the reduced model (without the non-significant three- and two-way interactions) testing the effect of relatedness, matrilineal-group membership and age difference on acoustic distance between dyads.

	Estimate	SE	df	Χ^2^	p value
(Intercept)	1.417	0.051			
relatedness	-0.012	0.009	1	1.704	0.192
age difference	0.198	0.022	1	53.913	<0.001
group-matrilineal membership:			2	8.774	0.012
same group- same matriline vs. different group- different matriline	-0.128	0.043	1	8.527	0.003
same group-different matriline vs. different group-different matriline	-0.067	0.044	1	2.203	0.138
same group-different matriline vs. same group-same matriline	0.057	0.034	1	2.776	0.096

**Fig 4:**
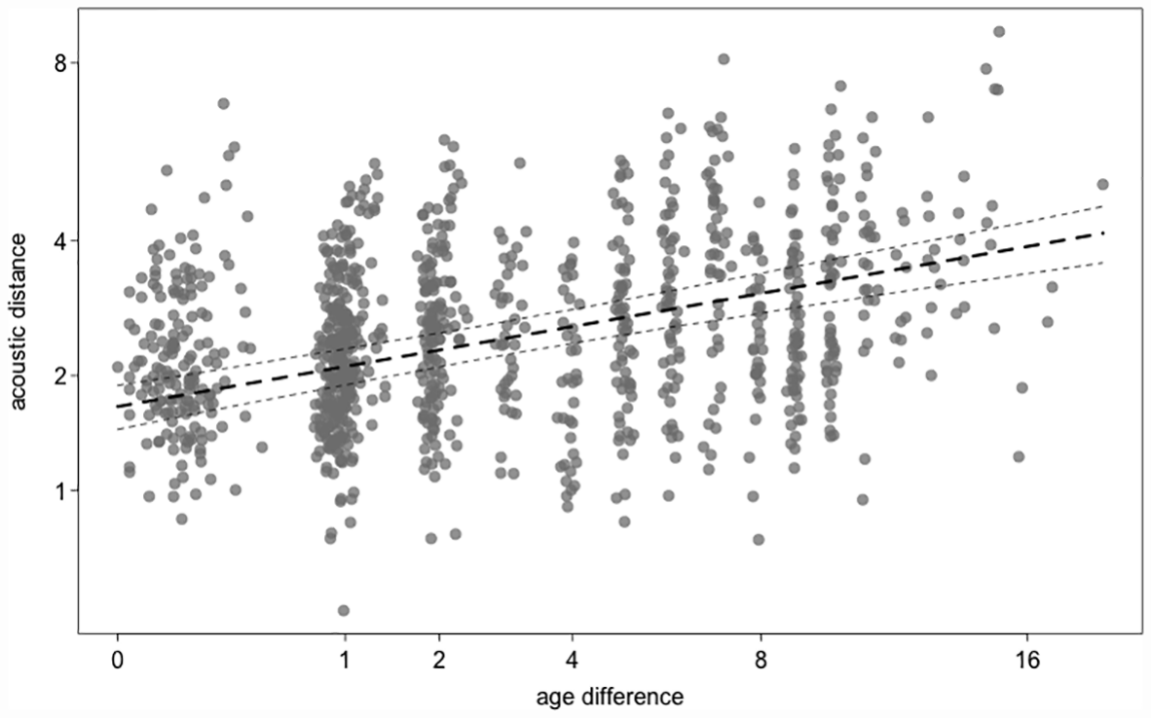
Acoustic distance vs. age difference. Acoustic distance plotted against age difference in years (at square root scale) within pairs of individuals (pooling the three levels of group-matrilineal membership). The dashed lines represent the model estimate for acoustic distance as a function of age difference (bold-dashed line) and the upper and lower confidence interval.

Surprisingly, we found that members of a dyad that had a similar age (i.e., smaller age difference) showed smaller acoustic distance/greater acoustic similarity (Table 3, Fig 4). This effect was independent of familiarity, i.e. whether subjects lived in the same or different matriline(s) or group(s), respectively. To check whether this result was due to subtle effects of relatedness, we repeated the analysis using calls from unrelated females only. As before, the analysis revealed no effect of familiarity on acoustic distance (LRT of full model including group-matrilineal membership interacting with age difference vs. reduced model excluding this interaction: Χ^2^ = 1.788, df = 2, P = 0.409). Thus, the greater acoustic similarity of dyads of similar age appeared to be due to overall changes with age, such that younger adult females collectively sounded different from older adult females (note that a dyad of greater age difference ultimately consisted of a younger and an older female).

## Discussion

Investigating contact calls of females of varying degrees of relatedness and familiarity (i.e., group-matrilineal membership and age difference), our analysis suggests familiarity but not relatedness to reflect acoustic distance within dyads. Specifically, females of similar age or females that grew up in the same group and matriline showed significant similarities in their ‘coo’ call structure. Such an effect of familiarity on call structure has been described previously. For instance, Snowdon and Elowson [53] reported that Pygmy marmosets (*Cebuella pygmaea*) modified their call structure when paired with new partners. Furthermore, free-living Campbell’s monkeys shared higher vocal similarity among closely bonded individuals, compared to individuals that hardly interacted [26]. That such a familiarity effect can also lead to similarities in vocal structures at the group level is suggested by a study on wild chimpanzees (*Pan troglodytes*) revealing temporal and structural differences in the same call type (the ‘pant hoot’) between two groups [54,55]. Furthermore, when presented with alarm calls of their own vs. another population, Barbary macaques (*Macaca sylvanus*) responded significantly longer toward the calls from the other groups [56]. Vocal accommodation seems very likely to explain the effect of familiarity found in this study, because non-human primates have only limited control over their vocal production [20,57].

In contrast to an effect of a high degree of familiarity, we could not detect significant similarities in ‘coo calls’ in relation to the degree of relatedness. This is a surprising result because a playback study conducted on the same population using the same call type showed that, independent of familiarity, females responded more often to ‘coo’ calls of paternal half-sisters than to ‘coo’ calls of unrelated females, suggesting acoustic phenotype matching [12]. On Cayo Santiago, however, inter-group encounters do occur, mostly around feeding sites. During such encounters, individuals might gain at least some knowledge about kin living in other social groups, for instance, via linking visual cues known to reflect kinship (see [13]) with vocal cues. In such case, the recognition of kin found in our playback study [12] could have been indirect, i.e., via the transfer of information about relatedness gained from one modality, e.g., vision, to a second modality, here vocal. However, since group encounters are mostly aggressive and, as such, are accompanied by agonistic vocalizations (e.g., barks, screams) rather than contact calls (i.e., coo’s), the extent to which such information transfer would be plausible is currently unknown and calls for more studies of this kind. Nevertheless, the idea that non-human primates can use phenotype matching in the vocal domain gains support in a recent playback study on mandrills, that also reported different response patterns (i.e., body and head movements towards the loudspeaker) in relation to genetic relatedness [14]. In the same study, the authors reported that kinship is reflected in the acoustic structure of the species’ contact calls [14]. However, the vocal characterization of individuals was based on call units rather than independently emitted calls, which precludes a direct comparison of our results with this study.

There are many factors that may affect the phenotypic expression of vocalizations, possibly masking vocal features that might reflect relatedness, one of which is vocal accommodation. Currently, there is no possibility to exclude that our structural analysis might have missed the acoustic feature(s) reflecting relatedness. However, the high number of females (67 subjects) included in our analysis as well as the high assignment quality of independently emitted ‘coo’ calls to individual subjects, 50 times above chance, makes it unlikely that we lacked statistical power to unravel possible structural similarities. In addition, the methodological procedure used in this study to estimate the similarity of calls, was used successfully in other studies. For instance, using the same score to calculate acoustic distance, a study on crested gibbons (*Namascus spp*.) showed a high concordance between genetic relatedness, geographic distance and acoustic structure of their songs [38]. In this study the acoustic distance score was sufficiently sensitive to unravel the phylogenetic relatedness of song structure, not only at species level but also at the level of a single population. A similar study on langurs (*Presbytis spp*.) comparing genetic relatedness, geographic distance and acoustic structure was likewise successful [39], confirming the utility of this methodological approach.

The advantage of our study design is the inclusion of paternal kin, in addition to maternal kin. Paternal kin are, due to the species migration regime, the only kin that potentially grew up and lived in different social groups, and hence, could be as unfamiliar to each other as possible in a natural setting. Given the prominent effect of familiarity on vocal structure, the number of unfamiliar paternal kin we were able to include into our analysis (19 unfamiliar paternal kin dyads vs. 249 unfamiliar non-kin dyads, see Table 1) compared to the number of familiar kin, however, might have made it difficult for analytical procedures to disentangle relatedness from familiarity (i.e., limited the power to detect on interaction between relatedness and familiarity).

Surprisingly, we found the effect of younger adult females sounding different from older adult females to be independent of familiarity, suggesting morphological changes in the sound apparatus as a likely source. So far, in adult females with a stable hierarchy, such changes have only been reported after dramatic modifications in hormone levels, such as, e.g., after entering menopause [58–60] or after hormone replacement therapy [61,62]. Since all our study subjects were still cycling with none being under hormonal contraception, another possible explanation for this effect are age related changes of tissue responsible for sound production (e.g., [63–66]).

In summary, our study provides evidence for familiarity, but not genetic relatedness, being reflected in the acoustic structure of rhesus macaque contact calls. While this result does not support phenotype matching as an underlying mechanism of kin recognition, it might be rooted in vocal accommodation. Further studies should focus on acoustic similarities between unfamiliar kin only, i.e., should fully control for the prominent effect of familiarity on the acoustic structure.

## Supporting Information

S1 Table Acoustic parameters used by the discriminant function analysis. Acoustic parameters representing the best combination to discriminate the 67 rhesus macaque females. Wilks-Lambda gives the reduction of total Wilks-Lambda by the entered variable.
(DOCX)

S2 Table Dataset. Data file containing necessary data to perform LMM analysis.
(PDF)
