# Supplementary material for: Does the Structure of Female Rhesus Macaque Coo Calls Reflect Relatedness and/or Familiarity?
Source: PLoS One. 2016 Aug 31;11(8):e0161133. doi: 10.1371/journal.pone.0161133 (PMC5007041; doi:10.1371/journal.pone.0161133)
Supplement: S1 Table — Acoustic parameters representing the best combination to discriminate the 67 rhesus macaque females. Wilks-Lambda gives the reduction of total Wilks-Lambda by the entered variable. (DOCX) [file pone.0161133.s001.docx]

S1 Table. Acoustic parameters representing the best combination to discriminate the 67 rhesus macaque females. Wilks-Lambda gives the reduction of total Wilks-Lambda by the entered variable.

|  | **acoustic features** | **Wilks-Lambda** | **description** |
| --- | --- | --- | --- |
| 1 | F0mean | 27.884 | Fundamental frequency (F0) mean across all time segments [Hz] |
| 2 | Duration | 14.396 | Duration of call [ms] |
| 3 | F0max | 10.649 | F0 maximum of all time segments [Hz] |
| 4 | Amptonal3 | 8.716 | Amplitude ration between 1st & 3rd harmonic |
| 5 | DF3mean | 7.474 | Mean frequency 3rd quartile DFA (distribution of frequency amplitude) [Hz] |
| 6 | DF1chmax | 6.677 | Maximum deviation between 1st DF and linear trend [Hz] |
| 7 | FP1mean | 6.085 | Start frequency of 1st global frequency peak [Hz] |
| 8 | F1mean | 5.591 | 1^st^ global frequency peak [Hz] |
| 9 | DF1chfreq | 5.201 | Number of changes between original and floating average curve (local modulation 1st DF) |
| 10 | DF1maxloc | 4.88 | [(1/duration)*location] |
| 11 | Ampratio1 | 4.613 | Amplitude ratio between 1st & 2nd DF |
| 12 | DF3% | 4.403 | Percentage of time segments with 3rd DF [%] |
| 13 | FP1amean | 4.227 | Mean amplitude of 1st GFP (difference to noise threshold) [rel. amplitude] |
| 14 | HNRmax | 4.072 | Maximum harmonic to noise ratio |
| 15 | PFmaxloc | 3.933 | [(1/duration)*location] |
| 16 | DFA3end | 3.802 | End frequency 3rd quartile DFA [Hz] |
| 17 | HNRmean | 3.686 | Mean harmonic to noise ratio |
| 18 | F1mran | 3.582 | Mean frequency range of 1st GFP [Hz] |
| 19 | FP1amax | 3.485 | Maximum amplitude of 1st GFP (difference to noise threshold) [rel. amplitude] |
| 20 | DF1fretr | 3.397 | Alternation frequency between 1st DF and linear trend |
| 21 | F0min | 3.318 | F0 minimum of all time segments [Hz] |
| 22 | Maxshimmer | 3.244 | Maximum of F0 amplitude modulation |
| 23 | DFfreq | 3.175 | Mean number of DF’s (dominant frequency bands) |
| 24 | F3% | 3.122 | Percentage of time segments with 3rd GFP [%] |
| 25 | DFA2min | 3.073 | Minimum frequency 2nd quartile DFA [Hz] |
| 26 | DFA3min | 3.02 | Minimum frequency 3rd quartile DFA [Hz] |
| 27 | Rangemax | 2.966 | Maximum frequency range of all time segments [Hz] |
| 28 | Disturb | 2.912 |  |
| 29 | PFmin | 2.862 | PF minimum of all time segments (peak frequency: highest frequency amplitude of a time segment) [Hz] |
| 30 | DF1st | 2.816 | Start frequency 1st DF (dominant frequency band) [Hz] |
| 31 | F2% | 2.77 | Percentage of time segments with 2nd GFP [%] |
| 32 | Rangemean | 2.743 | Mean frequency range across all time segments [Hz] |
| 33 | Ampratio3 | 2.701 | Amplitude ratio between 2nd & 3rd DF |
| 34 | Ampratio2 | 2.661 | Amplitude ratio between 1st & 3rd DF |
| 35 | DF3med | 2.624 | Median frequency 3rd DF [Hz] |
| 36 | PFtonmin | 2.589 | PF minimum of all tonal time segments |
| 37 | PFslope | 2.555 | Factor of linear trend of PF |
| 38 | PFmiloc | 2.524 | Location of the minimum PF [(1/duration)*location] |
| 39 | Diffmean | 2.494 | Mean difference between 1st & 2nd DF [Hz] |
| 40 | Rangetonm | 2.465 | Mean frequency range across all tonal time segments [Hz] |
| 41 | Meanjitter | 2.437 | Mean of F0 modulation |
| 42 | FP1max | 2.409 | Maximum frequency at GFP maximum [Hz] |
| 43 | PFmed | 2.385 | Median PF [Hz] |
| 44 | DFA1maloc | 2.361 | End frequency 3rd quartile DFA [Hz] |
| 45 | DFA1min | 2.337 | Minimum frequency 1st quartile DFA [Hz] |
| 46 | DFA1end | 2.319 | End frequency 1st quartile DFA [Hz] |
| 47 | DFA1med | 2.3 | Median frequency 1st quartile DFA [Hz] |
| 48 | F1maxran | 2.279 | Maximum frequency range of 1st GFP [Hz] |
| 49 | F1endran | 2.263 | End frequency range of 1st GFP [Hz] |
