# Supplementary material for: Does the Structure of Female Rhesus Macaque Coo Calls Reflect Relatedness and/or Familiarity?
Source: PLoS One. 2016 Aug 31;11(8):e0161133. doi: 10.1371/journal.pone.0161133 (PMC5007041; doi:10.1371/journal.pone.0161133)
Supplement: S2 Table — Data file containing necessary data to perform LMM analysis. (PDF) [file pone.0161133.s002.pdf]

	row.names	row_no	tierB	tierA	tab_R.tot	tab_pat.certain	tab_mat.certain	R	no.Vorfahren	sameMatriline	matriline.Tier.A
1	1	1	AAA	AAC	ja	ja	ja	0.0000	12	0	DM
2	3	3	AAA	AAG	ja	ja	ja	0.0000	12	0	DM
3	4	4	AAA	AAH	ja	ja	ja	0.0000	12	0	DM
4	5	5	AAA	AAJ	ja	ja	ja	0.0000	12	0	DM
5	6	6	AAA	AAL	ja	ja	ja	0.0000	12	0	DM
6	7	7	AAA	AAM	ja	ja	ja	0.0000	12	0	116
7	8	8	AAA	AAN	ja	ja	ja	0.0000	12	0	DM
8	9	9	AAA	AAO	ja	ja	ja	0.0000	12	0	DM
9	10	10	AAA	AAR	ja	nein	nein	0.1250	12	0	4
10	11	11	AAA	AAS	ja	ja	ja	0.0000	12	0	91
11	12	12	AAA	AAT	ja	ja	ja	0.0000	12	0	DM
12	13	13	AAA	AAU	ja	ja	ja	0.0000	12	0	DM
13	14	14	AAA	AAX	ja	ja	ja	0.0000	12	0	DM
14	15	15	AAA	AAY	ja	ja	ja	0.0000	12	0	DM
15	16	16	AAA	AAZ	ja	ja	ja	0.0000	12	0	91
16	17	17	AAA	ABB	ja	nein	nein	0.2500	11	0	4
17	18	18	AAA	ABC	ja	ja	ja	0.0000	12	0	DM
18	19	19	AAA	ABF	ja	ja	ja	0.0000	12	0	DM
19	20	20	AAA	ABG	ja	ja	ja	0.0000	12	0	91
20	21	21	AAA	ABH	ja	ja	ja	0.0000	12	0	91
21	22	22	AAA	ABI	ja	ja	ja	0.0000	12	0	DM
22	23	23	AAA	ABJ	ja	ja	ja	0.0000	12	0	DM
23	24	24	AAA	ABK	ja	nein	nein	0.5000	12	1	22
24	25	25	AAA	ABO	ja	ja	nein	0.0625	12	0	DM
25	26	26	AAA	ABP	ja	ja	ja	0.0000	12	0	DM
26	27	27	AAA	ABS	ja	ja	ja	0.0000	12	0	116
27	28	28	AAA	ABT	ja	ja	ja	0.0000	12	0	DM
28	29	29	AAA	ABU	ja	ja	ja	0.0000	12	0	65
29	30	30	AAA	ABV	ja	ja	ja	0.0000	12	0	116
30	31	31	AAA	ABW	ja	ja	ja	0.0000	12	0	91
31	32	32	AAA	ABY	ja	ja	ja	0.0000	12	0	DM
32	33	33	AAA	ABZ	ja	ja	ja	0.0000	12	0	DM
33	34	34	AAA	ACA	ja	ja	ja	0.0000	12	0	DM
34	35	35	AAA	ACC	ja	ja	ja	0.0000	12	0	DM
35	36	36	AAA	ACD	ja	ja	ja	0.0000	12	0	116
36	37	37	AAA	ACF	ja	ja	ja	0.0000	12	0	DM
37	38	38	AAA	ACG	ja	ja	ja	0.0000	12	0	DM
38	39	39	AAA	ACH	ja	ja	ja	0.0000	12	0	DM
39	40	40	AAA	ACI	ja	ja	ja	0.0000	12	0	116
40	41	41	AAA	ACJ	ja	ja	ja	0.0000	12	0	DM
41	42	42	AAA	ACK	ja	ja	ja	0.0000	12	0	DM
42	43	43	AAA	ACL	ja	ja	ja	0.0000	12	0	DM
43	44	44	AAB	AAL	ja	nein	nein	0.1250	10	1	DM
44	46	46	AAB	AAU	ja	nein	nein	0.1250	10	1	DM
45	47	47	AAB	AAZ	ja	nein	nein	0.1250	10	0	91
46	49	49	AAB	ABF	ja	nein	nein	0.5000	10	1	DM
47	50	50	AAB	ABI	ja	nein	nein	0.1250	10	1	DM
48	53	53	AAB	ABY	ja	nein	nein	0.1250	10	1	DM
49	54	54	AAB	ACC	ja	nein	nein	0.1250	10	1	DM
50	55	55	AAB	ACG	ja	nein	nein	0.5000	10	1	DM
51	56	56	AAB	ACH	ja	nein	nein	0.1250	10	1	DM
52	62	62	AAC	AAF	ja	nein	nein	0.2500	11	1	DM
53	63	63	AAC	AAG	ja	ja	ja	0.0000	12	1	DM
54	64	64	AAC	AAH	ja	ja	ja	0.0000	12	1	DM
55	65	65	AAC	AAJ	ja	nein	ja	0.0625	12	1	DM
56	66	66	AAC	AAL	ja	ja	ja	0.0000	12	1	DM
57	67	67	AAC	AAM	ja	ja	ja	0.0000	12	0	116
58	68	68	AAC	AAN	ja	nein	nein	0.1250	12	1	DM
59	69	69	AAC	AAO	ja	ja	nein	0.2500	12	1	DM
60	70	70	AAC	AAP	ja	nein	nein	0.1250	10	1	DM
61	71	71	AAC	AAR	ja	ja	ja	0.0000	12	0	4
62	72	72	AAC	AAS	ja	ja	ja	0.0000	12	0	91
63	73	73	AAC	AAT	ja	ja	ja	0.0000	12	1	DM
64	74	74	AAC	AAU	ja	nein	nein	0.1250	12	1	DM
65	75	75	AAC	AAV	ja	nein	nein	0.0625	11	1	DM

66	76	76	AAC	AAX	ja	ja	ja	0.0000	12	1	DM
67	77	77	AAC	AAY	ja	nein	nein	0.3125	12	1	DM
68	78	78	AAC	AAZ	ja	ja	ja	0.0000	12	0	91
69	79	79	AAC	ABC	ja	ja	ja	0.0000	12	1	DM
70	80	80	AAC	ABF	ja	ja	ja	0.0000	12	1	DM
71	81	81	AAC	ABG	ja	ja	ja	0.0000	12	0	91
72	82	82	AAC	ABH	ja	ja	ja	0.0000	12	0	91
73	83	83	AAC	ABI	ja	ja	nein	0.2500	12	1	DM
74	84	84	AAC	ABJ	ja	nein	nein	0.1250	12	1	DM
75	85	85	AAC	ABK	ja	ja	ja	0.0000	12	0	22
76	86	86	AAC	ABO	ja	ja	ja	0.0000	12	1	DM
77	87	87	AAC	ABP	ja	ja	ja	0.0000	12	1	DM
78	88	88	AAC	ABS	ja	ja	ja	0.0000	12	0	116
79	89	89	AAC	ABT	ja	ja	ja	0.0000	12	1	DM
80	90	90	AAC	ABU	ja	ja	ja	0.0000	12	0	65
81	91	91	AAC	ABV	ja	ja	ja	0.0000	12	0	116
82	92	92	AAC	ABW	ja	ja	ja	0.0000	12	0	91
83	93	93	AAC	ABX	ja	nein	nein	0.2500	11	1	DM
84	94	94	AAC	ABY	ja	ja	ja	0.0000	12	1	DM
85	95	95	AAC	ABZ	ja	ja	ja	0.0000	12	1	DM
86	96	96	AAC	ACA	ja	ja	ja	0.0000	12	1	DM
87	97	97	AAC	ACB	ja	nein	nein	0.0625	11	1	DM
88	98	98	AAC	ACC	ja	ja	nein	0.2500	12	1	DM
89	99	99	AAC	ACD	ja	ja	ja	0.0000	12	0	116
90	100	100	AAC	ACF	ja	ja	ja	0.0000	12	1	DM
91	101	101	AAC	ACG	ja	ja	ja	0.0000	12	1	DM
92	102	102	AAC	ACH	ja	ja	ja	0.0000	12	1	DM
93	103	103	AAC	ACI	ja	ja	ja	0.0000	12	0	116
94	104	104	AAC	ACJ	ja	ja	nein	0.1250	12	1	DM
95	105	105	AAC	ACK	ja	ja	ja	0.0000	12	1	DM
96	106	106	AAC	ACL	ja	ja	ja	0.0000	12	1	DM
97	107	107	AAC	ACP	ja	nein	nein	0.1250	11	1	DM
98	119	119	AAD	AAX	ja	ja	nein	0.0625	11	1	DM
99	125	125	AAD	ABH	ja	nein	nein	0.1250	11	0	91
100	135	135	AAD	ABW	ja	ja	nein	0.2500	11	0	91
101	136	136	AAD	ABY	ja	ja	nein	0.0625	11	1	DM
102	149	149	AAE	AAK	ja	nein	nein	0.2500	10	1	91
103	150	150	AAE	AAS	ja	nein	nein	0.0625	11	1	91
104	152	152	AAE	AAZ	ja	nein	nein	0.0625	11	1	91
105	153	153	AAE	ABE	ja	nein	nein	0.0625	10	0	DM
106	154	154	AAE	ABG	ja	nein	nein	0.2500	11	1	91
107	155	155	AAE	ABN	ja	nein	nein	0.0625	10	1	91
108	156	156	AAE	ABR	ja	nein	nein	0.1250	10	1	91
109	157	157	AAE	ABW	ja	nein	nein	0.0625	11	1	91
110	159	159	AAF	AAN	ja	nein	nein	0.1250	11	1	DM
111	160	160	AAF	AAO	ja	nein	nein	0.2500	11	1	DM
112	162	162	AAF	AAU	ja	nein	nein	0.1250	11	1	DM
113	163	163	AAF	AAY	ja	nein	nein	0.2500	11	1	DM
114	164	164	AAF	ABI	ja	nein	nein	0.2500	11	1	DM
115	165	165	AAF	ABJ	ja	nein	nein	0.1250	11	1	DM
116	166	166	AAF	ABX	ja	nein	nein	0.2500	10	1	DM
117	167	167	AAF	ACC	ja	nein	nein	0.2500	11	1	DM
118	168	168	AAF	ACJ	ja	nein	nein	0.1250	11	1	DM
119	170	170	AAG	AAH	ja	ja	ja	0.0000	12	1	DM
120	171	171	AAG	AAJ	ja	ja	ja	0.0000	12	1	DM
121	172	172	AAG	AAL	ja	nein	ja	0.1250	12	1	DM
122	173	173	AAG	AAM	ja	ja	ja	0.0000	12	0	116
123	174	174	AAG	AAN	ja	ja	ja	0.0000	12	1	DM
124	175	175	AAG	AAO	ja	ja	ja	0.0000	12	1	DM
125	176	176	AAG	AAR	ja	ja	ja	0.0000	12	0	4
126	177	177	AAG	AAS	ja	ja	nein	0.2500	12	0	91
127	178	178	AAG	AAT	ja	ja	ja	0.0000	12	1	DM
128	179	179	AAG	AAU	ja	ja	ja	0.0000	12	1	DM
129	180	180	AAG	AAX	ja	ja	ja	0.0000	12	1	DM
130	181	181	AAG	AAY	ja	ja	ja	0.0000	12	1	DM

131	182	182	AAG	AAZ	ja	ja	nein	0.0625	12	0	91
132	183	183	AAG	ABA	ja	nein	nein	0.2500	11	0	116
133	184	184	AAG	ABC	ja	ja	ja	0.0000	12	1	DM
134	185	185	AAG	ABF	ja	ja	ja	0.0000	12	1	DM
135	186	186	AAG	ABG	ja	ja	ja	0.0000	12	0	91
136	187	187	AAG	ABH	ja	ja	ja	0.0000	12	0	91
137	188	188	AAG	ABI	ja	ja	ja	0.0000	12	1	DM
138	189	189	AAG	ABJ	ja	ja	ja	0.0000	12	1	DM
139	190	190	AAG	ABK	ja	ja	ja	0.0000	12	0	22
140	191	191	AAG	ABL	ja	nein	nein	0.2500	11	1	DM
141	192	192	AAG	ABO	ja	ja	ja	0.0000	12	1	DM
142	193	193	AAG	ABP	ja	ja	nein	0.2500	12	1	DM
143	194	194	AAG	ABS	ja	ja	ja	0.0000	12	0	116
144	195	195	AAG	ABT	ja	ja	ja	0.0000	12	1	DM
145	196	196	AAG	ABU	ja	ja	ja	0.0000	12	0	65
146	197	197	AAG	ABV	ja	ja	ja	0.0000	12	0	116
147	198	198	AAG	ABW	ja	ja	ja	0.0000	12	0	91
148	199	199	AAG	ABY	ja	ja	ja	0.0000	12	1	DM
149	200	200	AAG	ABZ	ja	ja	nein	0.2500	12	1	DM
150	201	201	AAG	ACA	ja	nein	ja	0.2500	12	1	DM
151	202	202	AAG	ACC	ja	ja	ja	0.0000	12	1	DM
152	203	203	AAG	ACD	ja	ja	ja	0.0000	12	0	116
153	204	204	AAG	ACF	ja	ja	ja	0.0000	12	1	DM
154	205	205	AAG	ACG	ja	ja	ja	0.0000	12	1	DM
155	206	206	AAG	ACH	ja	nein	ja	0.5000	12	1	DM
156	207	207	AAG	ACI	ja	ja	ja	0.0000	12	0	116
157	208	208	AAG	ACJ	ja	ja	ja	0.0000	12	1	DM
158	209	209	AAG	ACK	ja	ja	ja	0.0000	12	1	DM
159	210	210	AAG	ACL	ja	ja	ja	0.0000	12	1	DM
160	211	211	AAG	ACQ	ja	nein	nein	0.1250	10	0	116
161	212	212	AAH	AAJ	ja	ja	ja	0.0000	12	1	DM
162	213	213	AAH	AAL	ja	nein	ja	0.0625	12	1	DM
163	214	214	AAH	AAM	ja	ja	ja	0.0000	12	0	116
164	215	215	AAH	AAN	ja	ja	ja	0.0000	12	1	DM
165	216	216	AAH	AAO	ja	ja	ja	0.0000	12	1	DM
166	217	217	AAH	AAR	ja	ja	ja	0.0000	12	0	4
167	218	218	AAH	AAS	ja	ja	ja	0.0000	12	0	91
168	219	219	AAH	AAT	ja	ja	ja	0.0000	12	1	DM
169	220	220	AAH	AAU	ja	ja	ja	0.0000	12	1	DM
170	221	221	AAH	AAX	ja	ja	ja	0.0000	12	1	DM
171	222	222	AAH	AAY	ja	ja	ja	0.0000	12	1	DM
172	223	223	AAH	AAZ	ja	ja	ja	0.0000	12	0	91
173	224	224	AAH	ABC	ja	ja	nein	0.2500	12	1	DM
174	225	225	AAH	ABF	ja	ja	ja	0.0000	12	1	DM
175	226	226	AAH	ABG	ja	ja	ja	0.0000	12	0	91
176	227	227	AAH	ABH	ja	ja	nein	0.2500	12	0	91
177	228	228	AAH	ABI	ja	ja	ja	0.0000	12	1	DM
178	229	229	AAH	ABJ	ja	ja	ja	0.0000	12	1	DM
179	230	230	AAH	ABK	ja	ja	ja	0.0000	12	0	22
180	231	231	AAH	ABL	ja	nein	nein	0.1250	11	1	DM
181	232	232	AAH	ABO	ja	ja	ja	0.0000	12	1	DM
182	233	233	AAH	ABP	ja	ja	ja	0.0000	12	1	DM
183	234	234	AAH	ABS	ja	ja	ja	0.0000	12	0	116
184	235	235	AAH	ABT	ja	ja	ja	0.0000	12	1	DM
185	236	236	AAH	ABU	ja	ja	ja	0.0000	12	0	65
186	237	237	AAH	ABV	ja	ja	ja	0.0000	12	0	116
187	238	238	AAH	ABW	ja	ja	ja	0.0000	12	0	91
188	239	239	AAH	ABY	ja	ja	ja	0.0000	12	1	DM
189	240	240	AAH	ABZ	ja	ja	ja	0.0000	12	1	DM
190	241	241	AAH	ACA	ja	ja	ja	0.0000	12	1	DM
191	242	242	AAH	ACC	ja	nein	ja	0.1250	12	1	DM
192	243	243	AAH	ACD	ja	ja	ja	0.0000	12	0	116
193	244	244	AAH	ACF	ja	ja	ja	0.0000	12	1	DM
194	245	245	AAH	ACG	ja	ja	ja	0.0000	12	1	DM
195	246	246	AAH	ACH	ja	nein	ja	0.0625	12	1	DM

196	247	247	AAH	ACI	ja	ja	ja	0.0000	12	0	116
197	248	248	AAH	ACJ	ja	ja	ja	0.0000	12	1	DM
198	249	249	AAH	ACK	ja	ja	nein	0.2500	12	1	DM
199	250	250	AAH	ACL	ja	ja	ja	0.0000	12	1	DM
200	251	251	AAH	ACM	ja	nein	nein	0.5000	11	1	DM
201	252	252	AAI	AAT	ja	nein	nein	0.1250	11	0	DM
202	253	253	AAI	ABA	ja	nein	nein	0.1250	10	1	116
203	256	256	AAJ	AAL	ja	ja	ja	0.0000	12	1	DM
204	257	257	AAJ	AAM	ja	ja	ja	0.0000	12	0	116
205	258	258	AAJ	AAN	ja	ja	ja	0.0000	12	1	DM
206	259	259	AAJ	AAO	ja	ja	ja	0.0000	12	1	DM
207	260	260	AAJ	AAP	ja	nein	nein	0.1250	10	1	DM
208	261	261	AAJ	AAR	ja	ja	ja	0.0000	12	0	4
209	262	262	AAJ	AAS	ja	ja	ja	0.0000	12	0	91
210	263	263	AAJ	AAT	ja	ja	ja	0.0000	12	1	DM
211	264	264	AAJ	AAU	ja	ja	ja	0.0000	12	1	DM
212	265	265	AAJ	AAV	ja	nein	nein	0.1250	11	1	DM
213	266	266	AAJ	AAX	ja	ja	ja	0.0000	12	1	DM
214	267	267	AAJ	AAY	ja	ja	ja	0.0000	12	1	DM
215	268	268	AAJ	AAZ	ja	ja	ja	0.0000	12	0	91
216	269	269	AAJ	ABC	ja	ja	ja	0.0000	12	1	DM
217	270	270	AAJ	ABF	ja	ja	ja	0.0000	12	1	DM
218	271	271	AAJ	ABG	ja	ja	ja	0.0000	12	0	91
219	272	272	AAJ	ABH	ja	ja	ja	0.0000	12	0	91
220	273	273	AAJ	ABI	ja	ja	ja	0.0000	12	1	DM
221	274	274	AAJ	ABJ	ja	ja	ja	0.0000	12	1	DM
222	275	275	AAJ	ABK	ja	ja	ja	0.0000	12	0	22
223	276	276	AAJ	ABO	ja	ja	ja	0.0000	12	1	DM
224	277	277	AAJ	ABP	ja	ja	ja	0.0000	12	1	DM
225	278	278	AAJ	ABS	ja	ja	ja	0.0000	12	0	116
226	279	279	AAJ	ABT	ja	ja	ja	0.0000	12	1	DM
227	280	280	AAJ	ABU	ja	ja	ja	0.0000	12	0	65
228	281	281	AAJ	ABV	ja	ja	ja	0.0000	12	0	116
229	282	282	AAJ	ABW	ja	ja	ja	0.0000	12	0	91
230	283	283	AAJ	ABY	ja	ja	ja	0.0000	12	1	DM
231	284	284	AAJ	ABZ	ja	ja	ja	0.0000	12	1	DM
232	285	285	AAJ	ACA	ja	ja	ja	0.0000	12	1	DM
233	286	286	AAJ	ACB	ja	nein	nein	0.0625	11	1	DM
234	287	287	AAJ	ACC	ja	ja	ja	0.0000	12	1	DM
235	288	288	AAJ	ACD	ja	ja	ja	0.0000	12	0	116
236	289	289	AAJ	ACF	ja	ja	ja	0.0000	12	1	DM
237	290	290	AAJ	ACG	ja	ja	ja	0.0000	12	1	DM
238	291	291	AAJ	ACH	ja	ja	ja	0.0000	12	1	DM
239	292	292	AAJ	ACI	ja	ja	ja	0.0000	12	0	116
240	293	293	AAJ	ACJ	ja	ja	ja	0.0000	12	1	DM
241	294	294	AAJ	ACK	ja	ja	ja	0.0000	12	1	DM
242	295	295	AAJ	ACL	ja	ja	ja	0.0000	12	1	DM
243	296	296	AAK	AAS	ja	nein	nein	0.0625	11	1	91
244	298	298	AAK	AAZ	ja	nein	nein	0.0625	11	1	91
245	299	299	AAK	ABE	ja	nein	nein	0.3125	10	0	DM
246	300	300	AAK	ABG	ja	nein	nein	0.2500	11	1	91
247	302	302	AAK	ABN	ja	nein	nein	0.0625	10	1	91
248	303	303	AAK	ABR	ja	nein	nein	0.1250	10	1	91
249	304	304	AAK	ABW	ja	nein	nein	0.0625	11	1	91
250	306	306	AAL	AAM	ja	ja	ja	0.0000	12	0	116
251	307	307	AAL	AAN	ja	ja	ja	0.0000	12	1	DM
252	308	308	AAL	AAO	ja	ja	ja	0.0000	12	1	DM
253	309	309	AAL	AAP	ja	nein	nein	0.1250	10	1	DM
254	310	310	AAL	AAR	ja	ja	ja	0.0000	12	0	4
255	311	311	AAL	AAS	ja	ja	ja	0.0000	12	0	91
256	312	312	AAL	AAT	ja	ja	ja	0.0000	12	1	DM
257	313	313	AAL	AAU	ja	ja	ja	0.0000	12	1	DM
258	314	314	AAL	AAX	ja	ja	ja	0.0000	12	1	DM
259	315	315	AAL	AAY	ja	ja	ja	0.0000	12	1	DM
260	316	316	AAL	AAZ	ja	nein	nein	0.0625	12	0	91

261	317	317	AAL	ABC	ja	ja	ja	0.0000	12	1	DM
262	318	318	AAL	ABF	ja	nein	nein	0.0625	12	1	DM
263	319	319	AAL	ABG	ja	ja	ja	0.0000	12	0	91
264	320	320	AAL	ABH	ja	ja	ja	0.0000	12	0	91
265	321	321	AAL	ABI	ja	nein	nein	0.0625	12	1	DM
266	322	322	AAL	ABJ	ja	ja	ja	0.0000	12	1	DM
267	323	323	AAL	ABK	ja	ja	ja	0.0000	12	0	22
268	324	324	AAL	ABL	ja	nein	nein	0.5000	11	1	DM
269	325	325	AAL	ABN	ja	nein	nein	0.0625	11	0	91
270	326	326	AAL	ABO	ja	ja	ja	0.0000	12	1	DM
271	327	327	AAL	ABP	ja	ja	ja	0.0000	12	1	DM
272	328	328	AAL	ABS	ja	ja	ja	0.0000	12	0	116
273	329	329	AAL	ABT	ja	ja	ja	0.0000	12	1	DM
274	330	330	AAL	ABU	ja	ja	ja	0.0000	12	0	65
275	331	331	AAL	ABV	ja	ja	ja	0.0000	12	0	116
276	332	332	AAL	ABW	ja	ja	ja	0.0000	12	0	91
277	333	333	AAL	ABY	ja	nein	nein	0.0625	12	1	DM
278	334	334	AAL	ABZ	ja	ja	ja	0.0000	12	1	DM
279	335	335	AAL	ACA	ja	nein	ja	0.1250	12	1	DM
280	336	336	AAL	ACC	ja	nein	ja	0.1250	12	1	DM
281	337	337	AAL	ACD	ja	ja	ja	0.0000	12	0	116
282	338	338	AAL	ACF	ja	ja	ja	0.0000	12	1	DM
283	339	339	AAL	ACG	ja	nein	nein	0.0625	12	1	DM
284	340	340	AAL	ACH	ja	nein	ja	0.2500	12	1	DM
285	341	341	AAL	ACI	ja	ja	ja	0.0000	12	0	116
286	342	342	AAL	ACJ	ja	ja	ja	0.0000	12	1	DM
287	343	343	AAL	ACK	ja	ja	ja	0.0000	12	1	DM
288	344	344	AAL	ACL	ja	ja	ja	0.0000	12	1	DM
289	345	345	AAL	ACM	ja	nein	nein	0.1250	11	1	DM
290	346	346	AAL	ACR	ja	nein	nein	0.1250	10	1	DM
291	347	347	AAL	ACS	ja	nein	nein	0.1250	10	0	91
292	348	348	AAM	AAN	ja	ja	ja	0.0000	12	0	DM
293	349	349	AAM	AAO	ja	ja	ja	0.0000	12	0	DM
294	350	350	AAM	AAR	ja	ja	ja	0.0000	12	0	4
295	351	351	AAM	AAS	ja	ja	ja	0.0000	12	0	91
296	352	352	AAM	AAT	ja	nein	nein	0.1250	12	0	DM
297	353	353	AAM	AAU	ja	ja	ja	0.0000	12	0	DM
298	354	354	AAM	AAX	ja	ja	ja	0.0000	12	0	DM
299	355	355	AAM	AAY	ja	ja	ja	0.0000	12	0	DM
300	356	356	AAM	AAZ	ja	ja	ja	0.0000	12	0	91
301	357	357	AAM	ABC	ja	ja	ja	0.0000	12	0	DM
302	358	358	AAM	ABF	ja	ja	ja	0.0000	12	0	DM
303	359	359	AAM	ABG	ja	nein	nein	0.1250	12	0	91
304	360	360	AAM	ABH	ja	ja	ja	0.0000	12	0	91
305	361	361	AAM	ABI	ja	ja	ja	0.0000	12	0	DM
306	362	362	AAM	ABJ	ja	ja	ja	0.0000	12	0	DM
307	363	363	AAM	ABK	ja	ja	ja	0.0000	12	0	22
308	364	364	AAM	ABO	ja	ja	ja	0.0000	12	0	DM
309	365	365	AAM	ABP	ja	ja	ja	0.0000	12	0	DM
310	366	366	AAM	ABS	ja	ja	ja	0.0000	12	1	116
311	367	367	AAM	ABT	ja	ja	ja	0.0000	12	0	DM
312	368	368	AAM	ABU	ja	ja	ja	0.0000	12	0	65
313	369	369	AAM	ABV	ja	nein	ja	0.5000	12	1	116
314	370	370	AAM	ABW	ja	ja	ja	0.0000	12	0	91
315	371	371	AAM	ABY	ja	ja	ja	0.0000	12	0	DM
316	372	372	AAM	ABZ	ja	ja	ja	0.0000	12	0	DM
317	373	373	AAM	ACA	ja	ja	ja	0.0000	12	0	DM
318	374	374	AAM	ACC	ja	ja	ja	0.0000	12	0	DM
319	375	375	AAM	ACD	ja	nein	ja	0.2500	12	1	116
320	376	376	AAM	ACF	ja	ja	ja	0.0000	12	0	DM
321	377	377	AAM	ACG	ja	ja	ja	0.0000	12	0	DM
322	378	378	AAM	ACH	ja	ja	nein	0.0625	12	0	DM
323	379	379	AAM	ACI	ja	ja	ja	0.0000	12	1	116
324	380	380	AAM	ACJ	ja	ja	ja	0.0000	12	0	DM
325	381	381	AAM	ACK	ja	ja	ja	0.0000	12	0	DM
326	382	382	AAM	ACL	ja	ja	ja	0.0000	12	0	DM

327	383	383	AAN	AAO	ja	nein	nein	0.1250	12	1	DM
328	384	384	AAN	AAR	ja	ja	ja	0.0000	12	0	4
329	385	385	AAN	AAS	ja	ja	ja	0.0000	12	0	91
330	386	386	AAN	AAT	ja	ja	ja	0.0000	12	1	DM
331	387	387	AAN	AAU	ja	nein	nein	0.0625	12	1	DM
332	388	388	AAN	AAX	ja	ja	ja	0.0000	12	1	DM
333	389	389	AAN	AAY	ja	nein	nein	0.1250	12	1	DM
334	390	390	AAN	AAZ	ja	ja	ja	0.0000	12	0	91
335	391	391	AAN	ABC	ja	ja	ja	0.0000	12	1	DM
336	392	392	AAN	ABF	ja	ja	ja	0.0000	12	1	DM
337	393	393	AAN	ABG	ja	ja	ja	0.0000	12	0	91
338	394	394	AAN	ABH	ja	ja	ja	0.0000	12	0	91
339	395	395	AAN	ABI	ja	nein	nein	0.1250	12	1	DM
340	396	396	AAN	ABJ	ja	nein	ja	0.2500	12	1	DM
341	397	397	AAN	ABK	ja	ja	ja	0.0000	12	0	22
342	398	398	AAN	ABO	ja	ja	ja	0.0000	12	1	DM
343	399	399	AAN	ABP	ja	ja	ja	0.0000	12	1	DM
344	400	400	AAN	ABS	ja	ja	nein	0.2500	12	0	116
345	401	401	AAN	ABT	ja	ja	ja	0.0000	12	1	DM
346	402	402	AAN	ABU	ja	ja	ja	0.0000	12	0	65
347	403	403	AAN	ABV	ja	ja	ja	0.0000	12	0	116
348	404	404	AAN	ABW	ja	ja	ja	0.0000	12	0	91
349	405	405	AAN	ABX	ja	nein	nein	0.5000	11	1	DM
350	406	406	AAN	ABY	ja	nein	ja	0.0625	12	1	DM
351	407	407	AAN	ABZ	ja	ja	ja	0.0000	12	1	DM
352	408	408	AAN	ACA	ja	ja	ja	0.0000	12	1	DM
353	409	409	AAN	ACC	ja	nein	nein	0.1250	12	1	DM
354	410	410	AAN	ACD	ja	ja	ja	0.0000	12	0	116
355	411	411	AAN	ACF	ja	ja	ja	0.0000	12	1	DM
356	412	412	AAN	ACG	ja	ja	ja	0.0000	12	1	DM
357	413	413	AAN	ACH	ja	ja	ja	0.0000	12	1	DM
358	414	414	AAN	ACI	ja	ja	nein	0.2500	12	0	116
359	415	415	AAN	ACJ	ja	nein	nein	0.0625	12	1	DM
360	416	416	AAN	ACK	ja	ja	ja	0.0000	12	1	DM
361	417	417	AAN	ACL	ja	nein	ja	0.0625	12	1	DM
362	418	418	AAN	ACR	ja	nein	nein	0.1250	10	1	DM
363	419	419	AAO	AAR	ja	ja	ja	0.0000	12	0	4
364	420	420	AAO	AAS	ja	ja	ja	0.0000	12	0	91
365	421	421	AAO	AAT	ja	nein	ja	0.1250	12	1	DM
366	422	422	AAO	AAU	ja	nein	nein	0.1250	12	1	DM
367	423	423	AAO	AAX	ja	ja	ja	0.0000	12	1	DM
368	424	424	AAO	AAY	ja	nein	nein	0.3750	12	1	DM
369	425	425	AAO	AAZ	ja	ja	ja	0.0000	12	0	91
370	426	426	AAO	ABC	ja	ja	ja	0.0000	12	1	DM
371	427	427	AAO	ABF	ja	ja	ja	0.0000	12	1	DM
372	428	428	AAO	ABG	ja	ja	ja	0.0000	12	0	91
373	429	429	AAO	ABH	ja	ja	ja	0.0000	12	0	91
374	430	430	AAO	ABI	ja	nein	nein	0.3125	12	1	DM
375	431	431	AAO	ABJ	ja	nein	nein	0.1250	12	1	DM
376	432	432	AAO	ABK	ja	ja	ja	0.0000	12	0	22
377	433	433	AAO	ABO	ja	ja	ja	0.0000	12	1	DM
378	434	434	AAO	ABP	ja	ja	ja	0.0000	12	1	DM
379	435	435	AAO	ABS	ja	ja	ja	0.0000	12	0	116
380	436	436	AAO	ABT	ja	ja	ja	0.0000	12	1	DM
381	437	437	AAO	ABU	ja	ja	ja	0.0000	12	0	65
382	438	438	AAO	ABV	ja	ja	ja	0.0000	12	0	116
383	439	439	AAO	ABW	ja	ja	ja	0.0000	12	0	91
384	440	440	AAO	ABX	ja	nein	nein	0.3125	11	1	DM
385	441	441	AAO	ABY	ja	ja	ja	0.0000	12	1	DM
386	442	442	AAO	ABZ	ja	ja	ja	0.0000	12	1	DM
387	443	443	AAO	ACA	ja	ja	ja	0.0000	12	1	DM
388	444	444	AAO	ACC	ja	ja	nein	0.2500	12	1	DM
389	445	445	AAO	ACD	ja	ja	ja	0.0000	12	0	116
390	446	446	AAO	ACF	ja	ja	ja	0.0000	12	1	DM
391	447	447	AAO	ACG	ja	ja	ja	0.0000	12	1	DM

392	448	448	AAO	ACH	ja	ja	ja	0.0000	12	1	DM
393	449	449	AAO	ACI	ja	ja	ja	0.0000	12	0	116
394	450	450	AAO	ACJ	ja	ja	nein	0.1250	12	1	DM
395	451	451	AAO	ACK	ja	ja	ja	0.0000	12	1	DM
396	452	452	AAO	ACL	ja	ja	ja	0.0000	12	1	DM
397	453	453	AAO	ACO	ja	nein	nein	0.0625	10	1	DM
398	454	454	AAO	ACP	ja	nein	nein	0.2500	11	1	DM
399	455	455	AAO	ACR	ja	nein	nein	0.0625	10	1	DM
400	457	457	AAP	AAZ	ja	nein	nein	0.1250	10	0	91
401	458	458	AAP	ABF	ja	nein	nein	0.1250	10	1	DM
402	459	459	AAP	ABI	ja	nein	nein	0.1250	10	1	DM
403	462	462	AAP	ABY	ja	nein	nein	0.1250	10	1	DM
404	464	464	AAP	ACG	ja	nein	nein	0.1250	10	1	DM
405	465	465	AAP	ACH	ja	nein	nein	0.1250	10	1	DM
406	469	469	AAQ	ACK	ja	nein	nein	0.1250	10	1	DM
407	471	471	AAR	AAS	ja	ja	ja	0.0000	12	0	91
408	472	472	AAR	AAT	ja	ja	ja	0.0000	12	0	DM
409	473	473	AAR	AAU	ja	ja	ja	0.0000	12	0	DM
410	474	474	AAR	AAX	ja	ja	ja	0.0000	12	0	DM
411	475	475	AAR	AAY	ja	ja	ja	0.0000	12	0	DM
412	476	476	AAR	AAZ	ja	ja	ja	0.0000	12	0	91
413	477	477	AAR	ABB	ja	nein	nein	0.5000	11	1	4
414	478	478	AAR	ABC	ja	ja	ja	0.0000	12	0	DM
415	479	479	AAR	ABF	ja	ja	ja	0.0000	12	0	DM
416	480	480	AAR	ABG	ja	ja	ja	0.0000	12	0	91
417	481	481	AAR	ABH	ja	ja	ja	0.0000	12	0	91
418	482	482	AAR	ABI	ja	ja	ja	0.0000	12	0	DM
419	483	483	AAR	ABJ	ja	ja	ja	0.0000	12	0	DM
420	484	484	AAR	ABK	ja	nein	nein	0.1250	12	0	22
421	485	485	AAR	ABO	ja	ja	ja	0.0000	12	0	DM
422	486	486	AAR	ABP	ja	ja	ja	0.0000	12	0	DM
423	487	487	AAR	ABS	ja	ja	ja	0.0000	12	0	116
424	488	488	AAR	ABT	ja	ja	ja	0.0000	12	0	DM
425	489	489	AAR	ABU	ja	ja	ja	0.0000	12	0	65
426	490	490	AAR	ABV	ja	ja	ja	0.0000	12	0	116
427	491	491	AAR	ABW	ja	ja	ja	0.0000	12	0	91
428	492	492	AAR	ABY	ja	ja	ja	0.0000	12	0	DM
429	493	493	AAR	ABZ	ja	ja	ja	0.0000	12	0	DM
430	494	494	AAR	ACA	ja	ja	ja	0.0000	12	0	DM
431	495	495	AAR	ACC	ja	ja	ja	0.0000	12	0	DM
432	496	496	AAR	ACD	ja	ja	ja	0.0000	12	0	116
433	497	497	AAR	ACF	ja	ja	ja	0.0000	12	0	DM
434	498	498	AAR	ACG	ja	ja	ja	0.0000	12	0	DM
435	499	499	AAR	ACH	ja	ja	ja	0.0000	12	0	DM
436	500	500	AAR	ACI	ja	ja	ja	0.0000	12	0	116
437	501	501	AAR	ACJ	ja	ja	ja	0.0000	12	0	DM
438	502	502	AAR	ACK	ja	ja	ja	0.0000	12	0	DM
439	503	503	AAR	ACL	ja	ja	ja	0.0000	12	0	DM
440	504	504	AAS	AAT	ja	ja	ja	0.0000	12	0	DM
441	505	505	AAS	AAU	ja	ja	ja	0.0000	12	0	DM
442	506	506	AAS	AAW	ja	nein	nein	0.1250	10	1	91
443	507	507	AAS	AAX	ja	ja	ja	0.0000	12	0	DM
444	508	508	AAS	AAY	ja	ja	ja	0.0000	12	0	DM
445	509	509	AAS	AAZ	ja	nein	nein	0.1250	12	1	91
446	510	510	AAS	ABA	ja	nein	nein	0.2500	11	0	116
447	511	511	AAS	ABC	ja	ja	ja	0.0000	12	0	DM
448	512	512	AAS	ABF	ja	ja	ja	0.0000	12	0	DM
449	513	513	AAS	ABG	ja	nein	ja	0.0625	12	1	91
450	514	514	AAS	ABH	ja	ja	ja	0.0000	12	1	91
451	515	515	AAS	ABI	ja	ja	ja	0.0000	12	0	DM
452	516	516	AAS	ABJ	ja	ja	ja	0.0000	12	0	DM
453	517	517	AAS	ABK	ja	ja	ja	0.0000	12	0	22
454	518	518	AAS	ABN	ja	nein	nein	0.0625	11	1	91
455	519	519	AAS	ABO	ja	ja	ja	0.0000	12	0	DM
456	520	520	AAS	ABP	ja	ja	nein	0.2500	12	0	DM

457	521	521	AAS	ABR	ja	nein	nein	0.5000	11	1	91
458	522	522	AAS	ABS	ja	ja	ja	0.0000	12	0	116
459	523	523	AAS	ABT	ja	ja	ja	0.0000	12	0	DM
460	524	524	AAS	ABU	ja	ja	ja	0.0000	12	0	65
461	525	525	AAS	ABV	ja	ja	ja	0.0000	12	0	116
462	526	526	AAS	ABW	ja	nein	ja	0.0625	12	1	91
463	527	527	AAS	ABY	ja	ja	ja	0.0000	12	0	DM
464	528	528	AAS	ABZ	ja	ja	nein	0.2500	12	0	DM
465	529	529	AAS	ACA	ja	ja	ja	0.0000	12	0	DM
466	530	530	AAS	ACC	ja	ja	ja	0.0000	12	0	DM
467	531	531	AAS	ACD	ja	nein	nein	0.1250	12	0	116
468	532	532	AAS	ACF	ja	ja	ja	0.0000	12	0	DM
469	533	533	AAS	ACG	ja	ja	ja	0.0000	12	0	DM
470	534	534	AAS	ACH	ja	ja	ja	0.0000	12	0	DM
471	535	535	AAS	ACI	ja	ja	ja	0.0000	12	0	116
472	536	536	AAS	ACJ	ja	ja	ja	0.0000	12	0	DM
473	537	537	AAS	ACK	ja	ja	ja	0.0000	12	0	DM
474	538	538	AAS	ACL	ja	ja	ja	0.0000	12	0	DM
475	539	539	AAS	ACQ	ja	nein	nein	0.1250	10	0	116
476	540	540	AAS	ACS	ja	nein	nein	0.1250	10	1	91
477	541	541	AAT	AAU	ja	ja	ja	0.0000	12	1	DM
478	542	542	AAT	AAX	ja	ja	ja	0.0000	12	1	DM
479	543	543	AAT	AAY	ja	nein	ja	0.0625	12	1	DM
480	544	544	AAT	AAZ	ja	ja	ja	0.0000	12	0	91
481	545	545	AAT	ABC	ja	ja	ja	0.0000	12	1	DM
482	546	546	AAT	ABF	ja	ja	ja	0.0000	12	1	DM
483	547	547	AAT	ABG	ja	ja	nein	0.2500	12	0	91
484	548	548	AAT	ABH	ja	ja	ja	0.0000	12	0	91
485	549	549	AAT	ABI	ja	ja	ja	0.0000	12	1	DM
486	550	550	AAT	ABJ	ja	ja	ja	0.0000	12	1	DM
487	551	551	AAT	ABK	ja	ja	ja	0.0000	12	0	22
488	552	552	AAT	ABO	ja	ja	ja	0.0000	12	1	DM
489	553	553	AAT	ABP	ja	ja	ja	0.0000	12	1	DM
490	554	554	AAT	ABS	ja	ja	ja	0.0000	12	0	116
491	555	555	AAT	ABT	ja	ja	ja	0.0000	12	1	DM
492	556	556	AAT	ABU	ja	ja	ja	0.0000	12	0	65
493	557	557	AAT	ABV	ja	ja	nein	0.2500	12	0	116
494	558	558	AAT	ABW	ja	ja	ja	0.0000	12	0	91
495	559	559	AAT	ABY	ja	ja	ja	0.0000	12	1	DM
496	560	560	AAT	ABZ	ja	ja	ja	0.0000	12	1	DM
497	561	561	AAT	ACA	ja	ja	ja	0.0000	12	1	DM
498	562	562	AAT	ACC	ja	ja	ja	0.0000	12	1	DM
499	563	563	AAT	ACD	ja	nein	nein	0.1250	12	0	116
500	564	564	AAT	ACF	ja	ja	ja	0.0000	12	1	DM
501	565	565	AAT	ACG	ja	ja	ja	0.0000	12	1	DM
502	566	566	AAT	ACH	ja	ja	ja	0.0000	12	1	DM
503	567	567	AAT	ACI	ja	ja	ja	0.0000	12	0	116
504	568	568	AAT	ACJ	ja	ja	ja	0.0000	12	1	DM
505	569	569	AAT	ACK	ja	ja	ja	0.0000	12	1	DM
506	570	570	AAT	ACL	ja	ja	ja	0.0000	12	1	DM
507	571	571	AAT	ACP	ja	nein	nein	0.1250	11	1	DM
508	572	572	AAU	AAX	ja	ja	ja	0.0000	12	1	DM
509	573	573	AAU	AAY	ja	nein	nein	0.1250	12	1	DM
510	574	574	AAU	AAZ	ja	ja	ja	0.0000	12	0	91
511	575	575	AAU	ABC	ja	ja	ja	0.0000	12	1	DM
512	576	576	AAU	ABE	ja	nein	nein	0.0625	11	1	DM
513	577	577	AAU	ABF	ja	nein	nein	0.0625	12	1	DM
514	578	578	AAU	ABG	ja	ja	ja	0.0000	12	0	91
515	579	579	AAU	ABH	ja	ja	ja	0.0000	12	0	91
516	580	580	AAU	ABI	ja	nein	ja	0.5000	12	1	DM
517	581	581	AAU	ABJ	ja	nein	nein	0.0625	12	1	DM
518	582	582	AAU	ABK	ja	ja	ja	0.0000	12	0	22
519	583	583	AAU	ABL	ja	nein	nein	0.0625	11	1	DM
520	584	584	AAU	ABO	ja	ja	ja	0.0000	12	1	DM
521	585	585	AAU	ABP	ja	ja	ja	0.0000	12	1	DM
522	586	586	AAU	ABS	ja	ja	ja	0.0000	12	0	116

523	587	587	AAU	ABT	ja	ja	ja	0.0000	12	1	DM
524	588	588	AAU	ABU	ja	ja	ja	0.0000	12	0	65
525	589	589	AAU	ABV	ja	ja	ja	0.0000	12	0	116
526	590	590	AAU	ABW	ja	ja	ja	0.0000	12	0	91
527	591	591	AAU	ABX	ja	nein	nein	0.1250	11	1	DM
528	592	592	AAU	ABY	ja	ja	ja	0.0000	12	1	DM
529	593	593	AAU	ABZ	ja	ja	ja	0.0000	12	1	DM
530	594	594	AAU	ACA	ja	ja	ja	0.0000	12	1	DM
531	595	595	AAU	ACC	ja	nein	nein	0.1875	12	1	DM
532	596	596	AAU	ACD	ja	ja	ja	0.0000	12	0	116
533	597	597	AAU	ACF	ja	ja	ja	0.0000	12	1	DM
534	598	598	AAU	ACG	ja	nein	nein	0.0625	12	1	DM
535	599	599	AAU	ACH	ja	ja	ja	0.0000	12	1	DM
536	600	600	AAU	ACI	ja	ja	ja	0.0000	12	0	116
537	601	601	AAU	ACJ	ja	nein	nein	0.0625	12	1	DM
538	602	602	AAU	ACK	ja	ja	ja	0.0000	12	1	DM
539	603	603	AAU	ACL	ja	ja	ja	0.0000	12	1	DM
540	604	604	AAU	ACM	ja	nein	nein	0.0625	11	1	DM
541	605	605	AAV	ABT	ja	nein	nein	0.5000	11	1	DM
542	606	606	AAV	ACB	ja	nein	nein	0.2500	10	1	DM
543	607	607	AAV	ACF	ja	nein	nein	0.1250	11	1	DM
544	608	608	AAW	AAZ	ja	nein	nein	0.1250	10	1	91
545	609	609	AAW	ABG	ja	nein	nein	0.1250	10	1	91
546	612	612	AAW	ABW	ja	nein	nein	0.1250	10	1	91
547	614	614	AAX	AAY	ja	ja	ja	0.0000	12	1	DM
548	615	615	AAX	AAZ	ja	ja	ja	0.0000	12	0	91
549	616	616	AAX	ABC	ja	ja	ja	0.0000	12	1	DM
550	617	617	AAX	ABF	ja	ja	ja	0.0000	12	1	DM
551	618	618	AAX	ABG	ja	ja	ja	0.0000	12	0	91
552	619	619	AAX	ABH	ja	ja	ja	0.0000	12	0	91
553	620	620	AAX	ABI	ja	ja	ja	0.0000	12	1	DM
554	621	621	AAX	ABJ	ja	ja	ja	0.0000	12	1	DM
555	622	622	AAX	ABK	ja	ja	ja	0.0000	12	0	22
556	623	623	AAX	ABO	ja	nein	ja	0.0625	12	1	DM
557	624	624	AAX	ABP	ja	nein	nein	0.1250	12	1	DM
558	625	625	AAX	ABS	ja	ja	ja	0.0000	12	0	116
559	626	626	AAX	ABT	ja	ja	ja	0.0000	12	1	DM
560	627	627	AAX	ABU	ja	ja	ja	0.0000	12	0	65
561	628	628	AAX	ABV	ja	ja	ja	0.0000	12	0	116
562	629	629	AAX	ABW	ja	ja	nein	0.0625	12	0	91
563	630	630	AAX	ABY	ja	ja	nein	0.2500	12	1	DM
564	631	631	AAX	ABZ	ja	ja	ja	0.0000	12	1	DM
565	632	632	AAX	ACA	ja	ja	ja	0.0000	12	1	DM
566	633	633	AAX	ACC	ja	ja	ja	0.0000	12	1	DM
567	634	634	AAX	ACD	ja	ja	ja	0.0000	12	0	116
568	635	635	AAX	ACF	ja	ja	ja	0.0000	12	1	DM
569	636	636	AAX	ACG	ja	ja	ja	0.0000	12	1	DM
570	637	637	AAX	ACH	ja	ja	ja	0.0000	12	1	DM
571	638	638	AAX	ACI	ja	ja	ja	0.0000	12	0	116
572	639	639	AAX	ACJ	ja	ja	ja	0.0000	12	1	DM
573	640	640	AAX	ACK	ja	ja	ja	0.0000	12	1	DM
574	641	641	AAX	ACL	ja	ja	ja	0.0000	12	1	DM
575	642	642	AAX	ACO	ja	nein	nein	0.2500	10	1	DM
576	643	643	AAY	AAZ	ja	ja	ja	0.0000	12	0	91
577	644	644	AAY	ABC	ja	ja	ja	0.0000	12	1	DM
578	645	645	AAY	ABF	ja	ja	ja	0.0000	12	1	DM
579	646	646	AAY	ABG	ja	ja	ja	0.0000	12	0	91
580	647	647	AAY	ABH	ja	ja	ja	0.0000	12	0	91
581	648	648	AAY	ABI	ja	ja	nein	0.2500	12	1	DM
582	649	649	AAY	ABJ	ja	nein	nein	0.1250	12	1	DM
583	650	650	AAY	ABK	ja	ja	ja	0.0000	12	0	22
584	651	651	AAY	ABO	ja	ja	ja	0.0000	12	1	DM
585	652	652	AAY	ABP	ja	ja	ja	0.0000	12	1	DM
586	653	653	AAY	ABS	ja	ja	ja	0.0000	12	0	116

587	654	654	AAY	ABT	ja	ja	ja	0.0000	12	1	DM
588	655	655	AAY	ABU	ja	ja	ja	0.0000	12	0	65
589	656	656	AAY	ABV	ja	ja	ja	0.0000	12	0	116
590	657	657	AAY	ABW	ja	ja	ja	0.0000	12	0	91
591	658	658	AAY	ABX	ja	nein	nein	0.2500	11	1	DM
592	659	659	AAY	ABY	ja	ja	ja	0.0000	12	1	DM
593	660	660	AAY	ABZ	ja	ja	ja	0.0000	12	1	DM
594	661	661	AAY	ACA	ja	ja	ja	0.0000	12	1	DM
595	662	662	AAY	ACC	ja	ja	nein	0.2500	12	1	DM
596	663	663	AAY	ACD	ja	ja	ja	0.0000	12	0	116
597	664	664	AAY	ACF	ja	ja	ja	0.0000	12	1	DM
598	665	665	AAY	ACG	ja	ja	ja	0.0000	12	1	DM
599	666	666	AAY	ACH	ja	ja	ja	0.0000	12	1	DM
600	667	667	AAY	ACI	ja	ja	ja	0.0000	12	0	116
601	668	668	AAY	ACJ	ja	ja	nein	0.1250	12	1	DM
602	669	669	AAY	ACK	ja	ja	ja	0.0000	12	1	DM
603	670	670	AAY	ACL	ja	ja	ja	0.0000	12	1	DM
604	671	671	AAY	ACP	ja	nein	nein	0.5000	11	1	DM
605	672	672	AAZ	ABA	ja	nein	nein	0.0625	11	0	116
606	673	673	AAZ	ABC	ja	ja	ja	0.0000	12	0	DM
607	674	674	AAZ	ABF	ja	nein	nein	0.0625	12	0	DM
608	675	675	AAZ	ABG	ja	nein	ja	0.0625	12	1	91
609	676	676	AAZ	ABH	ja	ja	ja	0.0000	12	1	91
610	677	677	AAZ	ABI	ja	nein	nein	0.0625	12	0	DM
611	678	678	AAZ	ABJ	ja	ja	ja	0.0000	12	0	DM
612	679	679	AAZ	ABK	ja	ja	ja	0.0000	12	0	22
613	680	680	AAZ	ABL	ja	nein	nein	0.1250	11	0	DM
614	681	681	AAZ	ABN	ja	nein	nein	0.2500	11	1	91
615	682	682	AAZ	ABO	ja	ja	ja	0.0000	12	0	DM
616	683	683	AAZ	ABP	ja	ja	nein	0.0625	12	0	DM
617	684	684	AAZ	ABR	ja	nein	nein	0.1250	11	1	91
618	685	685	AAZ	ABS	ja	ja	ja	0.0000	12	0	116
619	686	686	AAZ	ABT	ja	ja	ja	0.0000	12	0	DM
620	687	687	AAZ	ABU	ja	ja	ja	0.0000	12	0	65
621	688	688	AAZ	ABV	ja	ja	ja	0.0000	12	0	116
622	689	689	AAZ	ABW	ja	nein	ja	0.0625	12	1	91
623	690	690	AAZ	ABY	ja	nein	nein	0.0625	12	0	DM
624	691	691	AAZ	ABZ	ja	ja	nein	0.0625	12	0	DM
625	692	692	AAZ	ACA	ja	ja	ja	0.0000	12	0	DM
626	693	693	AAZ	ACC	ja	ja	ja	0.0000	12	0	DM
627	694	694	AAZ	ACD	ja	ja	ja	0.0000	12	0	116
628	695	695	AAZ	ACF	ja	ja	ja	0.0000	12	0	DM
629	696	696	AAZ	ACG	ja	nein	nein	0.0625	12	0	DM
630	697	697	AAZ	ACH	ja	nein	nein	0.0625	12	0	DM
631	698	698	AAZ	ACI	ja	ja	ja	0.0000	12	0	116
632	699	699	AAZ	ACJ	ja	nein	nein	0.1250	12	0	DM
633	700	700	AAZ	ACK	ja	ja	ja	0.0000	12	0	DM
634	701	701	AAZ	ACL	ja	ja	ja	0.0000	12	0	DM
635	702	702	AAZ	ACQ	ja	nein	nein	0.1250	10	0	116
636	703	703	AAZ	ACR	ja	nein	nein	0.1250	10	0	DM
637	704	704	AAZ	ACS	ja	nein	nein	0.5000	10	1	91
638	706	706	ABA	ABP	ja	nein	nein	0.2500	11	0	DM
639	707	707	ABA	ABZ	ja	nein	nein	0.2500	11	0	DM
640	709	709	ABB	ABK	ja	nein	nein	0.2500	11	0	22
641	710	710	ABB	ABO	ja	nein	nein	0.0625	11	0	DM
642	711	711	ABC	ABF	ja	ja	ja	0.0000	12	1	DM
643	712	712	ABC	ABG	ja	ja	ja	0.0000	12	0	91
644	713	713	ABC	ABH	ja	ja	nein	0.2500	12	0	91
645	714	714	ABC	ABI	ja	ja	ja	0.0000	12	1	DM
646	715	715	ABC	ABJ	ja	ja	ja	0.0000	12	1	DM
647	716	716	ABC	ABK	ja	ja	ja	0.0000	12	0	22
648	717	717	ABC	ABO	ja	ja	ja	0.0000	12	1	DM
649	718	718	ABC	ABP	ja	ja	ja	0.0000	12	1	DM
650	719	719	ABC	ABS	ja	ja	ja	0.0000	12	0	116
651	720	720	ABC	ABT	ja	ja	ja	0.0000	12	1	DM

652	721	721	ABC	ABU	ja	ja	ja	0.0000	12	0	65
653	722	722	ABC	ABV	ja	ja	ja	0.0000	12	0	116
654	723	723	ABC	ABW	ja	ja	ja	0.0000	12	0	91
655	724	724	ABC	ABY	ja	ja	ja	0.0000	12	1	DM
656	725	725	ABC	ABZ	ja	ja	ja	0.0000	12	1	DM
657	726	726	ABC	ACA	ja	ja	ja	0.0000	12	1	DM
658	727	727	ABC	ACC	ja	ja	ja	0.0000	12	1	DM
659	728	728	ABC	ACD	ja	ja	ja	0.0000	12	0	116
660	729	729	ABC	ACF	ja	ja	ja	0.0000	12	1	DM
661	730	730	ABC	ACG	ja	ja	ja	0.0000	12	1	DM
662	731	731	ABC	ACH	ja	ja	ja	0.0000	12	1	DM
663	732	732	ABC	ACI	ja	ja	ja	0.0000	12	0	116
664	733	733	ABC	ACJ	ja	ja	ja	0.0000	12	1	DM
665	734	734	ABC	ACK	ja	ja	nein	0.2500	12	1	DM
666	735	735	ABC	ACL	ja	ja	ja	0.0000	12	1	DM
667	736	736	ABE	ABF	ja	nein	nein	0.0625	11	1	DM
668	737	737	ABE	ABG	ja	nein	nein	0.0625	11	0	91
669	738	738	ABE	ABL	ja	nein	nein	0.0625	10	1	DM
670	740	740	ABE	ACC	ja	nein	nein	0.0625	11	1	DM
671	741	741	ABE	ACG	ja	nein	nein	0.0625	11	1	DM
672	742	742	ABE	ACM	ja	nein	nein	0.0625	10	1	DM
673	743	743	ABF	ABG	ja	ja	ja	0.0000	12	0	91
674	744	744	ABF	ABH	ja	nein	nein	0.0625	12	0	91
675	745	745	ABF	ABI	ja	nein	nein	0.0625	12	1	DM
676	746	746	ABF	ABJ	ja	ja	ja	0.0000	12	1	DM
677	747	747	ABF	ABK	ja	ja	ja	0.0000	12	0	22
678	748	748	ABF	ABL	ja	nein	nein	0.1875	11	1	DM
679	749	749	ABF	ABN	ja	nein	nein	0.0625	11	0	91
680	750	750	ABF	ABO	ja	ja	ja	0.0000	12	1	DM
681	751	751	ABF	ABP	ja	ja	ja	0.0000	12	1	DM
682	752	752	ABF	ABS	ja	ja	ja	0.0000	12	0	116
683	753	753	ABF	ABT	ja	ja	nein	0.2500	12	1	DM
684	754	754	ABF	ABU	ja	ja	ja	0.0000	12	0	65
685	755	755	ABF	ABV	ja	ja	ja	0.0000	12	0	116
686	756	756	ABF	ABW	ja	nein	nein	0.1250	12	0	91
687	757	757	ABF	ABY	ja	nein	nein	0.0625	12	1	DM
688	758	758	ABF	ABZ	ja	ja	ja	0.0000	12	1	DM
689	759	759	ABF	ACA	ja	ja	ja	0.0000	12	1	DM
690	760	760	ABF	ACB	ja	nein	nein	0.2500	11	1	DM
691	761	761	ABF	ACC	ja	nein	ja	0.0625	12	1	DM
692	762	762	ABF	ACD	ja	ja	ja	0.0000	12	0	116
693	763	763	ABF	ACF	ja	ja	ja	0.0000	12	1	DM
694	764	764	ABF	ACG	ja	nein	ja	0.2500	12	1	DM
695	765	765	ABF	ACH	ja	nein	nein	0.0625	12	1	DM
696	766	766	ABF	ACI	ja	ja	ja	0.0000	12	0	116
697	767	767	ABF	ACJ	ja	ja	ja	0.0000	12	1	DM
698	768	768	ABF	ACK	ja	ja	ja	0.0000	12	1	DM
699	769	769	ABF	ACL	ja	ja	ja	0.0000	12	1	DM
700	770	770	ABF	ACM	ja	nein	nein	0.0625	11	1	DM
701	771	771	ABF	ACR	ja	nein	nein	0.1250	10	1	DM
702	772	772	ABF	ACS	ja	nein	nein	0.1250	10	0	91
703	773	773	ABG	ABH	ja	ja	ja	0.0000	12	1	91
704	774	774	ABG	ABI	ja	ja	ja	0.0000	12	0	DM
705	775	775	ABG	ABJ	ja	ja	ja	0.0000	12	0	DM
706	776	776	ABG	ABK	ja	ja	ja	0.0000	12	0	22
707	777	777	ABG	ABN	ja	nein	nein	0.0625	11	1	91
708	778	778	ABG	ABO	ja	ja	ja	0.0000	12	0	DM
709	779	779	ABG	ABP	ja	ja	ja	0.0000	12	0	DM
710	780	780	ABG	ABR	ja	nein	nein	0.1250	11	1	91
711	781	781	ABG	ABS	ja	ja	ja	0.0000	12	0	116
712	782	782	ABG	ABT	ja	ja	ja	0.0000	12	0	DM
713	783	783	ABG	ABU	ja	ja	ja	0.0000	12	0	65
714	784	784	ABG	ABV	ja	ja	nein	0.2500	12	0	116
715	785	785	ABG	ABW	ja	nein	ja	0.0625	12	1	91
716	786	786	ABG	ABY	ja	ja	ja	0.0000	12	0	DM
717	787	787	ABG	ABZ	ja	ja	ja	0.0000	12	0	DM

718	788	788	ABG	ACA	ja	ja	ja	0.0000	12	0	DM
719	789	789	ABG	ACC	ja	ja	ja	0.0000	12	0	DM
720	790	790	ABG	ACD	ja	nein	nein	0.1250	12	0	116
721	791	791	ABG	ACF	ja	ja	ja	0.0000	12	0	DM
722	792	792	ABG	ACG	ja	ja	ja	0.0000	12	0	DM
723	793	793	ABG	ACH	ja	ja	ja	0.0000	12	0	DM
724	794	794	ABG	ACI	ja	ja	ja	0.0000	12	0	116
725	795	795	ABG	ACJ	ja	ja	ja	0.0000	12	0	DM
726	796	796	ABG	ACK	ja	ja	ja	0.0000	12	0	DM
727	797	797	ABG	ACL	ja	ja	ja	0.0000	12	0	DM
728	798	798	ABG	ACS	ja	nein	nein	0.1250	10	1	91
729	799	799	ABH	ABI	ja	ja	ja	0.0000	12	0	DM
730	800	800	ABH	ABJ	ja	ja	ja	0.0000	12	0	DM
731	801	801	ABH	ABK	ja	ja	ja	0.0000	12	0	22
732	802	802	ABH	ABO	ja	ja	ja	0.0000	12	0	DM
733	803	803	ABH	ABP	ja	ja	ja	0.0000	12	0	DM
734	804	804	ABH	ABS	ja	ja	ja	0.0000	12	0	116
735	805	805	ABH	ABT	ja	nein	nein	0.0625	12	0	DM
736	806	806	ABH	ABU	ja	ja	ja	0.0000	12	0	65
737	807	807	ABH	ABV	ja	ja	ja	0.0000	12	0	116
738	808	808	ABH	ABW	ja	nein	ja	0.5000	12	1	91
739	809	809	ABH	ABY	ja	ja	ja	0.0000	12	0	DM
740	810	810	ABH	ABZ	ja	ja	ja	0.0000	12	0	DM
741	811	811	ABH	ACA	ja	ja	ja	0.0000	12	0	DM
742	812	812	ABH	ACB	ja	nein	nein	0.0625	11	0	DM
743	813	813	ABH	ACC	ja	ja	ja	0.0000	12	0	DM
744	814	814	ABH	ACD	ja	ja	ja	0.0000	12	0	116
745	815	815	ABH	ACF	ja	ja	ja	0.0000	12	0	DM
746	816	816	ABH	ACG	ja	ja	ja	0.0000	12	0	DM
747	817	817	ABH	ACH	ja	ja	ja	0.0000	12	0	DM
748	818	818	ABH	ACI	ja	ja	ja	0.0000	12	0	116
749	819	819	ABH	ACJ	ja	ja	ja	0.0000	12	0	DM
750	820	820	ABH	ACK	ja	ja	nein	0.2500	12	0	DM
751	821	821	ABH	ACL	ja	ja	ja	0.0000	12	0	DM
752	822	822	ABI	ABJ	ja	nein	nein	0.1250	12	1	DM
753	823	823	ABI	ABK	ja	ja	ja	0.0000	12	0	22
754	824	824	ABI	ABL	ja	nein	nein	0.1250	11	1	DM
755	825	825	ABI	ABN	ja	nein	nein	0.0625	11	0	91
756	826	826	ABI	ABO	ja	ja	ja	0.0000	12	1	DM
757	827	827	ABI	ABP	ja	ja	ja	0.0000	12	1	DM
758	828	828	ABI	ABS	ja	ja	ja	0.0000	12	0	116
759	829	829	ABI	ABT	ja	ja	ja	0.0000	12	1	DM
760	830	830	ABI	ABU	ja	ja	ja	0.0000	12	0	65
761	831	831	ABI	ABV	ja	ja	ja	0.0000	12	0	116
762	832	832	ABI	ABW	ja	ja	ja	0.0000	12	0	91
763	833	833	ABI	ABX	ja	nein	nein	0.3125	11	1	DM
764	834	834	ABI	ABY	ja	nein	nein	0.0625	12	1	DM
765	835	835	ABI	ABZ	ja	ja	ja	0.0000	12	1	DM
766	836	836	ABI	ACA	ja	ja	ja	0.0000	12	1	DM
767	837	837	ABI	ACC	ja	ja	nein	0.2500	12	1	DM
768	838	838	ABI	ACD	ja	ja	ja	0.0000	12	0	116
769	839	839	ABI	ACF	ja	ja	ja	0.0000	12	1	DM
770	840	840	ABI	ACG	ja	nein	nein	0.0625	12	1	DM
771	841	841	ABI	ACH	ja	nein	nein	0.0625	12	1	DM
772	842	842	ABI	ACI	ja	ja	ja	0.0000	12	0	116
773	843	843	ABI	ACJ	ja	ja	nein	0.1250	12	1	DM
774	844	844	ABI	ACK	ja	ja	ja	0.0000	12	1	DM
775	845	845	ABI	ACL	ja	ja	ja	0.0000	12	1	DM
776	846	846	ABI	ACO	ja	nein	nein	0.0625	10	1	DM
777	847	847	ABI	ACP	ja	nein	nein	0.0625	11	1	DM
778	848	848	ABI	ACR	ja	nein	nein	0.1875	10	1	DM
779	849	849	ABI	ACS	ja	nein	nein	0.1250	10	0	91
780	850	850	ABJ	ABK	ja	ja	ja	0.0000	12	0	22
781	851	851	ABJ	ABO	ja	ja	ja	0.0000	12	1	DM
782	852	852	ABJ	ABP	ja	ja	ja	0.0000	12	1	DM

783	853	853	ABJ	ABS	ja	ja	ja	0.0000	12	0	116
784	854	854	ABJ	ABT	ja	ja	ja	0.0000	12	1	DM
785	855	855	ABJ	ABU	ja	ja	ja	0.0000	12	0	65
786	856	856	ABJ	ABV	ja	ja	ja	0.0000	12	0	116
787	857	857	ABJ	ABW	ja	ja	ja	0.0000	12	0	91
788	858	858	ABJ	ABX	ja	nein	nein	0.5000	11	1	DM
789	859	859	ABJ	ABY	ja	nein	ja	0.0625	12	1	DM
790	860	860	ABJ	ABZ	ja	ja	ja	0.0000	12	1	DM
791	861	861	ABJ	ACA	ja	ja	ja	0.0000	12	1	DM
792	862	862	ABJ	ACC	ja	nein	nein	0.1250	12	1	DM
793	863	863	ABJ	ACD	ja	ja	ja	0.0000	12	0	116
794	864	864	ABJ	ACF	ja	ja	ja	0.0000	12	1	DM
795	865	865	ABJ	ACG	ja	ja	ja	0.0000	12	1	DM
796	866	866	ABJ	ACH	ja	ja	ja	0.0000	12	1	DM
797	867	867	ABJ	ACI	ja	ja	ja	0.0000	12	0	116
798	868	868	ABJ	ACJ	ja	nein	nein	0.0625	12	1	DM
799	869	869	ABJ	ACK	ja	ja	ja	0.0000	12	1	DM
800	870	870	ABJ	ACL	ja	nein	ja	0.0625	12	1	DM
801	871	871	ABJ	ACR	ja	nein	nein	0.1250	10	1	DM
802	872	872	ABK	ABO	ja	ja	nein	0.0625	12	0	DM
803	873	873	ABK	ABP	ja	ja	ja	0.0000	12	0	DM
804	874	874	ABK	ABS	ja	ja	ja	0.0000	12	0	116
805	875	875	ABK	ABT	ja	ja	ja	0.0000	12	0	DM
806	876	876	ABK	ABU	ja	ja	ja	0.0000	12	0	65
807	877	877	ABK	ABV	ja	ja	ja	0.0000	12	0	116
808	878	878	ABK	ABW	ja	ja	ja	0.0000	12	0	91
809	879	879	ABK	ABY	ja	ja	ja	0.0000	12	0	DM
810	880	880	ABK	ABZ	ja	ja	ja	0.0000	12	0	DM
811	881	881	ABK	ACA	ja	ja	ja	0.0000	12	0	DM
812	882	882	ABK	ACC	ja	ja	ja	0.0000	12	0	DM
813	883	883	ABK	ACD	ja	ja	ja	0.0000	12	0	116
814	884	884	ABK	ACF	ja	ja	ja	0.0000	12	0	DM
815	885	885	ABK	ACG	ja	ja	ja	0.0000	12	0	DM
816	886	886	ABK	ACH	ja	ja	ja	0.0000	12	0	DM
817	887	887	ABK	ACI	ja	ja	ja	0.0000	12	0	116
818	888	888	ABK	ACJ	ja	ja	ja	0.0000	12	0	DM
819	889	889	ABK	ACK	ja	ja	ja	0.0000	12	0	DM
820	890	890	ABK	ACL	ja	ja	ja	0.0000	12	0	DM
821	891	891	ABL	ABN	ja	nein	nein	0.1250	10	0	91
822	892	892	ABL	ABY	ja	nein	nein	0.1250	11	1	DM
823	893	893	ABL	ACA	ja	nein	nein	0.2500	11	1	DM
824	894	894	ABL	ACC	ja	nein	nein	0.2500	11	1	DM
825	895	895	ABL	ACG	ja	nein	nein	0.1875	11	1	DM
826	896	896	ABL	ACH	ja	nein	nein	0.5000	11	1	DM
827	897	897	ABL	ACM	ja	nein	nein	0.2500	10	1	DM
828	901	901	ABN	ABR	ja	nein	nein	0.1250	10	1	91
829	902	902	ABN	ABW	ja	nein	nein	0.0625	11	1	91
830	903	903	ABN	ABY	ja	nein	nein	0.0625	11	0	DM
831	904	904	ABN	ACE	ja	nein	nein	0.0625	10	0	65
832	905	905	ABN	ACG	ja	nein	nein	0.0625	11	0	DM
833	906	906	ABN	ACH	ja	nein	nein	0.0625	11	0	DM
834	907	907	ABN	ACJ	ja	nein	nein	0.1250	11	0	DM
835	910	910	ABO	ABP	ja	ja	ja	0.0000	12	1	DM
836	911	911	ABO	ABS	ja	ja	ja	0.0000	12	0	116
837	912	912	ABO	ABT	ja	ja	ja	0.0000	12	1	DM
838	913	913	ABO	ABU	ja	ja	ja	0.0000	12	0	65
839	914	914	ABO	ABV	ja	ja	ja	0.0000	12	0	116
840	915	915	ABO	ABW	ja	ja	ja	0.0000	12	0	91
841	916	916	ABO	ABY	ja	ja	ja	0.0000	12	1	DM
842	917	917	ABO	ABZ	ja	ja	ja	0.0000	12	1	DM
843	918	918	ABO	ACA	ja	ja	ja	0.0000	12	1	DM
844	919	919	ABO	ACC	ja	ja	ja	0.0000	12	1	DM
845	920	920	ABO	ACD	ja	ja	ja	0.0000	12	0	116
846	921	921	ABO	ACF	ja	ja	ja	0.0000	12	1	DM
847	922	922	ABO	ACG	ja	ja	ja	0.0000	12	1	DM
848	923	923	ABO	ACH	ja	ja	ja	0.0000	12	1	DM

849	924	924	ABO	ACI	ja	ja	ja	0.0000	12	0	116
850	925	925	ABO	ACJ	ja	ja	ja	0.0000	12	1	DM
851	926	926	ABO	ACK	ja	ja	ja	0.0000	12	1	DM
852	927	927	ABO	ACL	ja	ja	ja	0.0000	12	1	DM
853	928	928	ABO	ACO	ja	nein	nein	0.2500	10	1	DM
854	929	929	ABP	ABS	ja	ja	ja	0.0000	12	0	116
855	930	930	ABP	ABT	ja	ja	ja	0.0000	12	1	DM
856	931	931	ABP	ABU	ja	ja	ja	0.0000	12	0	65
857	932	932	ABP	ABV	ja	ja	ja	0.0000	12	0	116
858	933	933	ABP	ABW	ja	ja	ja	0.0000	12	0	91
859	934	934	ABP	ABY	ja	nein	ja	0.5000	12	1	DM
860	935	935	ABP	ABZ	ja	ja	nein	0.2500	12	1	DM
861	936	936	ABP	ACA	ja	ja	ja	0.0000	12	1	DM
862	937	937	ABP	ACC	ja	ja	ja	0.0000	12	1	DM
863	938	938	ABP	ACD	ja	ja	ja	0.0000	12	0	116
864	939	939	ABP	ACF	ja	ja	ja	0.0000	12	1	DM
865	940	940	ABP	ACG	ja	ja	ja	0.0000	12	1	DM
866	941	941	ABP	ACH	ja	ja	ja	0.0000	12	1	DM
867	942	942	ABP	ACI	ja	ja	ja	0.0000	12	0	116
868	943	943	ABP	ACJ	ja	ja	ja	0.0000	12	1	DM
869	944	944	ABP	ACK	ja	ja	ja	0.0000	12	1	DM
870	945	945	ABP	ACL	ja	ja	ja	0.0000	12	1	DM
871	946	946	ABP	ACQ	ja	nein	nein	0.1250	10	0	116
872	947	947	ABP	ACR	ja	nein	nein	0.2500	10	1	DM
873	949	949	ABR	ABV	ja	nein	nein	0.0625	11	0	116
874	950	950	ABR	ABW	ja	nein	nein	0.1250	11	1	91
875	951	951	ABR	ACD	ja	nein	nein	0.2500	11	0	116
876	953	953	ABS	ABT	ja	ja	ja	0.0000	12	0	DM
877	954	954	ABS	ABU	ja	ja	ja	0.0000	12	0	65
878	955	955	ABS	ABV	ja	ja	ja	0.0000	12	1	116
879	956	956	ABS	ABW	ja	ja	ja	0.0000	12	0	91
880	957	957	ABS	ABY	ja	ja	ja	0.0000	12	0	DM
881	958	958	ABS	ABZ	ja	ja	ja	0.0000	12	0	DM
882	959	959	ABS	ACA	ja	ja	ja	0.0000	12	0	DM
883	960	960	ABS	ACC	ja	ja	ja	0.0000	12	0	DM
884	961	961	ABS	ACD	ja	ja	ja	0.0000	12	1	116
885	962	962	ABS	ACF	ja	nein	nein	0.0625	12	0	DM
886	963	963	ABS	ACG	ja	ja	ja	0.0000	12	0	DM
887	964	964	ABS	ACH	ja	ja	ja	0.0000	12	0	DM
888	965	965	ABS	ACI	ja	ja	nein	0.2500	12	1	116
889	966	966	ABS	ACJ	ja	ja	ja	0.0000	12	0	DM
890	967	967	ABS	ACK	ja	ja	ja	0.0000	12	0	DM
891	968	968	ABS	ACL	ja	ja	ja	0.0000	12	0	DM
892	969	969	ABT	ABU	ja	ja	ja	0.0000	12	0	65
893	970	970	ABT	ABV	ja	ja	ja	0.0000	12	0	116
894	971	971	ABT	ABW	ja	nein	nein	0.1250	12	0	91
895	972	972	ABT	ABY	ja	ja	ja	0.0000	12	1	DM
896	973	973	ABT	ABZ	ja	ja	ja	0.0000	12	1	DM
897	974	974	ABT	ACA	ja	ja	ja	0.0000	12	1	DM
898	975	975	ABT	ACB	ja	nein	nein	0.3750	11	1	DM
899	976	976	ABT	ACC	ja	ja	ja	0.0000	12	1	DM
900	977	977	ABT	ACD	ja	ja	ja	0.0000	12	0	116
901	978	978	ABT	ACF	ja	nein	ja	0.0625	12	1	DM
902	979	979	ABT	ACG	ja	ja	ja	0.0000	12	1	DM
903	980	980	ABT	ACH	ja	ja	ja	0.0000	12	1	DM
904	981	981	ABT	ACI	ja	ja	ja	0.0000	12	0	116
905	982	982	ABT	ACJ	ja	ja	ja	0.0000	12	1	DM
906	983	983	ABT	ACK	ja	ja	ja	0.0000	12	1	DM
907	984	984	ABT	ACL	ja	ja	ja	0.0000	12	1	DM
908	985	985	ABU	ABV	ja	ja	ja	0.0000	12	0	116
909	986	986	ABU	ABW	ja	ja	ja	0.0000	12	0	91
910	987	987	ABU	ABY	ja	ja	ja	0.0000	12	0	DM
911	988	988	ABU	ABZ	ja	ja	ja	0.0000	12	0	DM
912	989	989	ABU	ACA	ja	ja	ja	0.0000	12	0	DM
913	990	990	ABU	ACC	ja	ja	ja	0.0000	12	0	DM

914	991	991	ABU	ACD	ja	ja	ja	0.0000	12	0	116
915	992	992	ABU	ACE	ja	nein	nein	0.5000	11	1	65
916	993	993	ABU	ACF	ja	ja	ja	0.0000	12	0	DM
917	994	994	ABU	ACG	ja	ja	ja	0.0000	12	0	DM
918	995	995	ABU	ACH	ja	ja	ja	0.0000	12	0	DM
919	996	996	ABU	ACI	ja	ja	ja	0.0000	12	0	116
920	997	997	ABU	ACJ	ja	ja	ja	0.0000	12	0	DM
921	998	998	ABU	ACK	ja	ja	ja	0.0000	12	0	DM
922	999	999	ABU	ACL	ja	ja	ja	0.0000	12	0	DM
923	1000	1000	ABV	ABW	ja	ja	ja	0.0000	12	0	91
924	1001	1001	ABV	ABY	ja	ja	ja	0.0000	12	0	DM
925	1002	1002	ABV	ABZ	ja	ja	ja	0.0000	12	0	DM
926	1003	1003	ABV	ACA	ja	ja	ja	0.0000	12	0	DM
927	1004	1004	ABV	ACC	ja	ja	ja	0.0000	12	0	DM
928	1005	1005	ABV	ACF	ja	ja	ja	0.0000	12	0	DM
929	1006	1006	ABV	ACG	ja	ja	ja	0.0000	12	0	DM
930	1007	1007	ABV	ACH	ja	ja	ja	0.0000	12	0	DM
931	1008	1008	ABV	ACI	ja	ja	ja	0.0000	12	1	116
932	1009	1009	ABV	ACJ	ja	ja	ja	0.0000	12	0	DM
933	1010	1010	ABV	ACK	ja	ja	ja	0.0000	12	0	DM
934	1011	1011	ABV	ACL	ja	ja	ja	0.0000	12	0	DM
935	1012	1012	ABW	ABY	ja	ja	nein	0.0625	12	0	DM
936	1013	1013	ABW	ABZ	ja	ja	ja	0.0000	12	0	DM
937	1014	1014	ABW	ACA	ja	ja	ja	0.0000	12	0	DM
938	1015	1015	ABW	ACB	ja	nein	nein	0.1250	11	0	DM
939	1016	1016	ABW	ACC	ja	ja	ja	0.0000	12	0	DM
940	1017	1017	ABW	ACD	ja	ja	ja	0.0000	12	0	116
941	1018	1018	ABW	ACF	ja	ja	ja	0.0000	12	0	DM
942	1019	1019	ABW	ACG	ja	ja	ja	0.0000	12	0	DM
943	1020	1020	ABW	ACH	ja	ja	ja	0.0000	12	0	DM
944	1021	1021	ABW	ACI	ja	ja	ja	0.0000	12	0	116
945	1022	1022	ABW	ACJ	ja	ja	ja	0.0000	12	0	DM
946	1023	1023	ABW	ACK	ja	ja	ja	0.0000	12	0	DM
947	1024	1024	ABW	ACL	ja	ja	ja	0.0000	12	0	DM
948	1025	1025	ABW	ACS	ja	nein	nein	0.1250	10	1	91
949	1026	1026	ABX	ABY	ja	nein	nein	0.1250	11	1	DM
950	1027	1027	ABX	ACC	ja	nein	nein	0.2500	11	1	DM
951	1028	1028	ABX	ACJ	ja	nein	nein	0.1250	11	1	DM
952	1029	1029	ABX	ACL	ja	nein	nein	0.1250	11	1	DM
953	1031	1031	ABX	ACP	ja	nein	nein	0.0625	10	1	DM
954	1033	1033	ABY	ABZ	ja	ja	ja	0.0000	12	1	DM
955	1034	1034	ABY	ACA	ja	ja	ja	0.0000	12	1	DM
956	1035	1035	ABY	ACC	ja	ja	ja	0.0000	12	1	DM
957	1036	1036	ABY	ACD	ja	ja	ja	0.0000	12	0	116
958	1037	1037	ABY	ACF	ja	ja	ja	0.0000	12	1	DM
959	1038	1038	ABY	ACG	ja	nein	nein	0.0625	12	1	DM
960	1039	1039	ABY	ACH	ja	nein	nein	0.0625	12	1	DM
961	1040	1040	ABY	ACI	ja	ja	ja	0.0000	12	0	116
962	1041	1041	ABY	ACJ	ja	ja	ja	0.0000	12	1	DM
963	1042	1042	ABY	ACK	ja	ja	ja	0.0000	12	1	DM
964	1043	1043	ABY	ACL	ja	nein	ja	0.0625	12	1	DM
965	1044	1044	ABY	ACR	ja	nein	nein	0.5000	10	1	DM
966	1045	1045	ABY	ACS	ja	nein	nein	0.1250	10	0	91
967	1046	1046	ABZ	ACA	ja	ja	ja	0.0000	12	1	DM
968	1047	1047	ABZ	ACC	ja	ja	ja	0.0000	12	1	DM
969	1048	1048	ABZ	ACD	ja	ja	ja	0.0000	12	0	116
970	1049	1049	ABZ	ACF	ja	ja	ja	0.0000	12	1	DM
971	1050	1050	ABZ	ACG	ja	ja	ja	0.0000	12	1	DM
972	1051	1051	ABZ	ACH	ja	ja	ja	0.0000	12	1	DM
973	1052	1052	ABZ	ACI	ja	ja	ja	0.0000	12	0	116
974	1053	1053	ABZ	ACJ	ja	ja	ja	0.0000	12	1	DM
975	1054	1054	ABZ	ACK	ja	ja	ja	0.0000	12	1	DM
976	1055	1055	ABZ	ACL	ja	ja	ja	0.0000	12	1	DM
977	1056	1056	ABZ	ACQ	ja	nein	nein	0.1250	10	0	116
978	1057	1057	ACA	ACC	ja	ja	ja	0.0000	12	1	DM

979	1058	1058	ACA	ACD	ja	ja	ja	0.0000	12	0	116
980	1059	1059	ACA	ACF	ja	ja	ja	0.0000	12	1	DM
981	1060	1060	ACA	ACG	ja	ja	ja	0.0000	12	1	DM
982	1061	1061	ACA	ACH	ja	nein	ja	0.5000	12	1	DM
983	1062	1062	ACA	ACI	ja	ja	ja	0.0000	12	0	116
984	1063	1063	ACA	ACJ	ja	ja	ja	0.0000	12	1	DM
985	1064	1064	ACA	ACK	ja	ja	ja	0.0000	12	1	DM
986	1065	1065	ACA	ACL	ja	ja	ja	0.0000	12	1	DM
987	1066	1066	ACB	ACF	ja	nein	nein	0.1250	11	1	DM
988	1067	1067	ACC	ACD	ja	ja	ja	0.0000	12	0	116
989	1068	1068	ACC	ACF	ja	ja	ja	0.0000	12	1	DM
990	1069	1069	ACC	ACG	ja	nein	ja	0.0625	12	1	DM
991	1070	1070	ACC	ACH	ja	nein	ja	0.1250	12	1	DM
992	1071	1071	ACC	ACI	ja	ja	ja	0.0000	12	0	116
993	1072	1072	ACC	ACJ	ja	ja	nein	0.1250	12	1	DM
994	1073	1073	ACC	ACK	ja	ja	ja	0.0000	12	1	DM
995	1074	1074	ACC	ACL	ja	ja	ja	0.0000	12	1	DM
996	1075	1075	ACC	ACM	ja	nein	nein	0.2500	11	1	DM
997	1076	1076	ACD	ACF	ja	ja	ja	0.0000	12	0	DM
998	1077	1077	ACD	ACG	ja	ja	ja	0.0000	12	0	DM
999	1078	1078	ACD	ACH	ja	ja	ja	0.0000	12	0	DM
1000	1079	1079	ACD	ACI	ja	ja	ja	0.0000	12	1	116
